# Adapting NDBIs for Autistic Children Who Use Echolalia: A Cascading Coaching Approach to Supporting Communication and Language Development

**DOI:** 10.3390/bs16091553

**Published:** 2026-09-02

**Authors:** Jessica Nico, Audriana Sauceda, Jaime Branaman, Cindy Gevarter

**Affiliations:** Department of Speech and Hearing Sciences, University of New Mexico, Albuquerque, NM 87131, USAjbranaman@unm.edu (J.B.); cgevarter@unm.edu (C.G.)

**Keywords:** autism, echolalia, naturalistic developmental behavioral intervention (NDBI), cascading coaching

## Abstract

This pilot study examined a cascading coaching model to train graduate student clinicians and caregivers to use adapted Naturalistic Developmental Behavioral Intervention (NDBI) techniques with autistic preschoolers who use echolalia. Adaptations were designed to incorporate children’s echolalic speech within intervention activities and support changes in spoken language. Strategies emphasized responding to, expanding upon, and creating opportunities around children’s verbal productions during play. Two clinician–caregiver–child triads participated. Effects were evaluated using multiple-probe designs across adult participants and intervention contexts. Across four weeks, adults increased their use of targeted NDBI skills, including elicitation techniques (e.g., creating opportunities, wait time, prompting) and response techniques (responsive actions and language models), with moderate to very large effects. Child participants also demonstrated increases in targeted spoken-language communication responses across intervention contexts, with the strongest effects observed during student clinician-led interactions and more variable outcomes during caregiver-led interactions. Visual analysis indicated functional relations for most adult and child outcomes, although individual differences and variability were observed across participants. Findings provide preliminary evidence regarding the feasibility of incorporating echolalia-responsive adaptations within NDBI-based coaching programs for autistic children who use echolalia and their caregivers.

## 1. Introduction

Naturalistic Developmental Behavioral Interventions (NDBIs) are evidence-based interventions that can be used to target a variety of outcomes for young autistic^1^ children (Han et al., 2025; Song et al., 2025). NDBIs capitalize on natural, motivating moments to create contextualized, meaningful learning opportunities within everyday social interactions (Schreibman et al., 2015). Although there are a variety of manualized NDBI programs such as the Early Start Denver Model (ESDM), Project ImPACT, and Joint Attention Symbolic Play Engagement and Regulation (JASPER), the core techniques embedded within these programs share common elements (Frost et al., 2020; Ousley et al., 2024). Generally, NDBIs that specifically target social communication skills follow a three-phase process: (a) what communication partners do before the child communicates (e.g., eliciting communication); (b) how the child attempts to communicate (e.g., child’s response); (c) what communication partners do after the child communicates (e.g., using related response techniques; Ousley et al., 2024). Intervention techniques common across NDBI approaches include promoting child engagement (e.g., face-to-face positioning, following the child’s lead, using positive affect), modeling developmentally appropriate language, and embedding direct teaching within natural interactions via elicitation techniques (e.g., temptations, wait time, prompting) and response techniques (e.g., natural reinforcement and responsive interactions; Frost et al., 2020).

Given the role of the child’s everyday communication partners in NDBIs, partner instruction (including structured caregiver coaching) is often embedded within the intervention process. Meta-analytic findings indicate that parent and caregiver-mediated NDBI interventions can be delivered with fidelity and yield meaningful improvements in children’s language outcomes (Ouyang et al., 2024). Emerging research also supports the effectiveness of cascading coaching models, in which instruction is delivered across tiers (e.g., researcher to clinician to caregiver), for improving adult fidelity and promoting child communication outcomes within NDBI frameworks (Gallegos et al., 2024; Gevarter et al., 2025; McGuire et al., 2025; Nattress et al., in press).

NDBIs have a well-established evidence base demonstrating positive intervention effects on generalized communication, expressive and receptive language, as well as social communication in autistic preschoolers (Han et al., 2025; Song et al., 2025). It is important to examine, however, the specific characteristics of autistic children and families who have been included in the literature. For instance, as NDBIs were developed primarily in Western middle-class contexts, minoritized children and families have often been excluded from research (Douglas et al., 2026). Although there is an emerging base of research that has embedded cultural adaptations within caregiver-led interventions, most often with Hispanic families, clear descriptions of adaptations and their effects are lacking (Douglas et al., 2026; McGuire et al., 2025).

Additionally, while several literature reviews have specifically focused on NDBIs for minimally verbal children diagnosed with autism (Pope et al., 2024; Ouyang et al., 2024), comparatively limited attention has been paid to children with autism spectrum disorder (ASD) who use spoken language but experience reduced intelligibility or difficulties generalizing spoken communication across communication partners and contexts. Research suggests that up to 34% of autistic children who use spoken language are only understood by familiar listeners (e.g., parents) rather than a wide range of individuals (e.g., peers, community members; Andzik et al., 2018). The existing evidence base has rarely reported the inclusion or exclusion of autistic children who use idiosyncratic verbal language patterns that limit generalization of communication skills. This includes, for example, children with autism who use echolalia, the behavior of repeating another person’s speech, or using previously heard phrases or sentences in apparently unrelated contexts.

### 1.1. Conceptualizing Echolalia in Autism

The observable behavior of repeating speech, either immediately or after a delay, is described using various terms across medical, developmental and behavioral disciplines, including “echolalia,” “vocal stereotypy,” and “verbal stereotypy”; however, terminology and operational definitions vary across disciplines, and there is limited consensus regarding how these behaviors should be defined and distinguished (Cohn et al., 2023a; McFayden et al., 2022; Stiegler, 2015). Developmentally, imitation and repetition skills are essential prelinguistic skills, and as such, are positive prognostic indicators within early language development (McFayden et al., 2022). Beyond the early language development stage, however, children with ASD demonstrate imitative speech at a significantly higher rate than their typically developing peers (Van Santen et al., 2013). Despite this knowledge, there is no accurate, contemporary estimate of echolalia use among autistic children and adolescents (Sutherland et al., 2024). Importantly, immediate echolalia (imitating a phrase, word, or sentence right after a model) and delayed echolalia (repeating a phrase sometime after it was heard) are considered “unconventional verbal behavior” (Stiegler, 2015, p. 751) and included as diagnostic indicators on the Autism Diagnostic Observation Schedule (ADOS; Lord et al., 2012).

Echolalia may present as a child’s arbitrary, acontextual, or atypical association between phrases and meanings (Lord et al., 2012). For example, an autistic child who uses echolalia may hear a phrase such as “get out of my car” from a movie, and then use that phrase in other contexts, such as when someone moves into their physical space. Alternatively, autistic children who use echolalia may overgeneralize commonly heard phrases/sentences that are limited in specificity (e.g., “I want this,” “Where is it?”). To a familiar listener or when contextual information is clearly apparent, the underlying communicative message of a child’s echolalia may be easily recognized. Parents of autistic children report that their ability to understand their child’s echolalic speech can vary significantly based upon structure, context, function, origin, time difference, and underlying meaning (Cohn et al., 2023a). Although some listeners may recognize and interpret the communicative intent underlying echolalic speech, this understanding may not be consistent across communication partners or contexts.

While familiar partners may be more likely to understand the intent behind an echolalic response (e.g., a child advocating for space), at other times, the child’s communicative message may not be easily understood. For autistic children who use echolalia, communication may be less effective when interacting with unfamiliar listeners, discussing unshared contexts, or using unclear referents (Cohn et al., 2023a, Cohn et al., 2024). As a result, the idiosyncratic meaning behind a child’s echolalic utterances may make them noncommunicative to most communication partners (Gleitman, 2017). Supporting autistic children who use echolalia in developing a broader range of communicative forms and functions through echolalia intervention may enhance successful communication across people, places, and activities.

A common assumption underlying existing echolalia intervention research is that echolalia is inherently pathological, automatic, meaningless, maladaptive, disruptive, or non-intentional (Blackburn et al., 2023; Ryan et al., 2022). However, the persistence of echolalic speech suggests that it serves a function for the autistic child, even when that function is not immediately apparent to communication partners. Existing echolalia intervention research often aims to reduce immediate and/or delayed echolalia, revealing inconsistent outcomes and little clarity as to whether this reduction improves meaningful communication for the child (Blackburn et al., 2023; Neely et al., 2016). Emerging literature around echolalia suggests it may not be an indicator of a deficit, but rather a different approach to communication, reciprocity, and social interaction (McFayden et al., 2022). Recent qualitative studies examining caregiver perspectives on echolalia suggest that it may be viewed not only as a language learning method or communication modality, but as an expression of neurodiversity and part of their child’s individual identity (Cohn et al., 2023a; Cohn et al., 2024). These views align with neurodiversity-affirming approaches to intervention which emphasize principles such as presuming competence, supporting communication across modalities, honoring sensory and regulatory needs, and fostering autonomy and self-determination (Donaldson et al., 2017). While NDBIs have been proposed as neurodiversity-affirming and strengths-based compatible approaches to early language intervention, adaptations and intentional planning are necessary to meet the needs of specific ASD populations, such as autistic children who use echolalia (Schuck et al., 2022; Wang et al., 2023).

### 1.2. Leveraging Echolalia Within NDBI Interventions

Several core features of NDBIs especially relevant for supporting autistic children with a history of echolalia include: (a) the individualization of communication goals and targets; (b) use of NDBI techniques and loose shaping procedures to reinforce closer approximations of targets; (c) the incorporation of child interests into intervention activities; (d) the integration of caregiver knowledge and lived experiences into the NDBI intervention process (Schreibman et al., 2015).

Communication outcomes for autistic children who use echolalia should be considered not only in terms of response frequency, but also in relation to broader dimensions of language development. Longstanding models of language assessment conceptualize language across the domains of form, content, and use (Lahey, 1988). Form refers to the structural characteristics of language, including syntax and utterance complexity; content refers to vocabulary and semantic specificity and use refers to communicative and pragmatic functions. These dimensions remain central to current speech-language pathology clinical practice (Wilder & Redmond, 2024) and provide a clinically relevant framework for understanding and evaluating communication outcomes among autistic children who use echolalia. Beyond early communication skills such as requesting and single-word utterances, NDBI techniques can be used to support more advanced language outcomes, including the expansion of communicative functions, utterance complexity, and vocabulary (Ousley et al., 2024). This may be particularly relevant for children who use echolalia, whose language often reflects unique patterns of form, vocabulary use, and communicative intent (Cohn et al., 2023a).

Echolalic speech may represent functional communication that could be shaped toward greater specificity, flexibility, and generalization across people, places, and activities. NDBI techniques such as individualized goal selection, responsive language modeling, and loose shaping may provide opportunities to support language development by expanding utterance complexity, increasing semantic specificity, and promoting a broader range of communicative functions within natural interactions. The identification of individualized communication targets allows clinicians to build upon existing echolalic utterances to support the production of longer, flexible sentence structures, expansion of expressive vocabulary, and incorporation of a broader range of communicative functions (e.g., directing, requesting assistance, or expressing problems). The principle of loose shaping, often used to target verbal language (Schreibman et al., 2015), can be used to reinforce echolalic, scripted, or idiosyncratic language. Through loose shaping, echolalic utterances can be framed as meaningful approximations of communication while gradually introducing more flexible syntax (form), specific or contextually matched vocabulary (content), or generalizable verbal operants (communicative functions).

Further individualization can be achieved by embedding preferred videos and activities within intervention and partnering with meaningful communication partners to inform goal selection, intervention planning, and responsive interaction strategies. Echolalic language drawn from a child’s favorite shows, YouTube videos, or movies can be leveraged and incorporated in NDBI. Additionally, caregivers of autistic children provide unique insights into the role of echolalia within their child’s communication and expression of neurodivergence (Cohn et al., 2023a, 2024). Inclusion of caregivers within an NDBI intervention and coaching program allows clinicians to incorporate caregivers’ existing knowledge about their child’s communication, which could support generalization of language structures, content and use across other communication partners or contexts. While these adaptations represent promising avenues for expanding the application of NDBIs to echolalic autistic children, no research to date has examined NDBI interventions specifically tailored to this population. As a result, clinicians and caregivers have limited evidence-based options for supporting children who use atypical language beyond early development.

### 1.3. Study Aims

To address these gaps in the echolalia and NDBI literature, the present study replicated and extended previous NDBI cascading coaching model research, which focused on training graduate student clinicians and caregivers to support the communication skills of autistic children who have minimal speech (Gallegos et al., 2024; Gevarter et al., 2025; McGuire et al., 2025; Nattress et al., in press). Specifically, this study examined speech-language pathology graduate student clinicians’ and caregivers’ use of NDBI communication techniques to expand targeted spoken communication skills of autistic children who use echolalia. Importantly, the present study examined child communication outcomes across both student clinician- and caregiver-led interactions, allowing for the evaluation of changes in communication responses across communication partners. Specifically, this study examined whether a brief NDBI cascading coaching program adapted for autistic children who use echolalia led to the following:

(a) increases in student clinicians’ and caregivers’ use of targeted NDBI *elicitation* (create and wait/prompt) and *response* techniques (responsive actions and responsive language models) for autistic preschoolers who use echolalia?
(b) increases in the use of targeted spoken communication responses (total and unprompted) among autistic preschoolers who use echolalia?

## 2. Methods and Materials

### 2.1. Participants 

Two triads (each consisting of an autistic child, their caregiver, and a graduate student clinician in speech and hearing sciences) participated in the study. Both child participants, Remy (triad 1) and Angel (triad 2), were autistic 4-year-old males, and their participating caregivers included Remy’s mother (Natalie) and Angel’s mother (Naomi). Graduate student clinicians included Emma (triad 1) and Liza (triad 2).

#### 2.1.1. Child Participants

Inclusion criteria for child participants in this study were as follows: (a) aged 2;11 through 5;0; (b) had an independent medical ASD diagnosis; (c) spoke English as a primary language output; (d) had a history of echolalia use as reported by parents and confirmed via clinical screening and play observation, and (e) were participating in a university-based free summer autism group clinic. Recruitment of caregivers and children was completed via outreach to local early intervention and preschool programs as well as referrals and collaboration with a university-based speech-language clinic, an autism assessment clinic, local private SLP providers, and parent Facebook groups. Once families contacted the researchers for participation within the autism summer clinic, potential child participants were screened against the inclusion criteria. Throughout screening sessions, the first author interviewed the mothers of child participants and observed each child during unstructured play to gather information used for screening and intervention planning. During unstructured play, each child interacted with an undergraduate or graduate student clinician who was told to offer the child a variety of toys and follow their lead during play (with researcher support as needed).

Child receptive language, expressive language, and interpersonal relationship skills were assessed via corresponding subtests within the Vineland Adaptive Behavior Scales 3rd Edition (VABS-III; Sparrow et al., 2016). A standardized measure specific to echolalic language is not yet available; therefore a combination of caregiver report, a researcher-developed echolalia questionnaire (see Supplementary Material for a refined version of the echolalia questionnaire adapted from Cohn et al., 2023a, 2023b; Mergl & Azoni, 2015), and naturalistic observation were utilized to determine a history of echolalia. This approach aligns with recommendations for triangulating direct assessment, parent questionnaire, and parent interview within ASD language assessment (Luyster et al., 2008). Given the lack of consensus regarding the definition and distinction of delayed echolalia in the literature, caregivers were asked the extent to which their child repeated speech immediately or after a period of time, rather than explicitly categorizing instances as delayed echolalia. For observation, researchers referred to operational definitions of ADOS items where echolalia is examined, noting presence of: (a) immediate echolalia and/or (b) stereotyped/idiosyncratic use of words or phrases, including delayed echolalia (Lord et al., 2012). While observing the child during unstructured, child-led play, the researcher documented field notes regarding: (a) child’s current level of play skills (e.g., play sequences within cause-effect play, functional play, pretend play, role play, etc.); (b) child interests; (c) current levels of contextualized language comprehension and production, (d) use of immediate or delayed echolalia and/or use of idiosyncratic or non-contextually matched phrases. Child sensory preferences and special interests were further documented via a parent interview using the Reinforcer Assessment for Individuals with Severe Disabilities (RAISD; Fisher et al., 1996, 2021). Table 1 provides an overview of child participants’ characteristics, with more detailed communication profiles described below.

Remy, an African-Canadian preschool-aged autistic boy, exposed to French, Yemba and English, had a caregiver-reported spoken vocabulary of more than 50 words. Remy and his family were recent immigrants to the United States. His expressive communication included early symbolic forms (e.g., pointing and gestures), simple syntactic structures (i.e., one- to three-word utterances), concrete vocabulary (e.g., colors, common objects, familiar people, early verbs), and a limited variety of early communicative functions, including rejecting, requesting objects, and labeling. Remy’s mother reported a history of echolalia. Although his expressive communication was not limited to echolalic speech, Remy frequently repeated words and phrases immediately following communication partner models and was observed recalling and producing previously modeled words, phrases, and echolalic utterances after a delay. Additionally, his mother reported frequent use of jargon-like utterances mixed with true words, as well as echolalic language across contexts. Remy attended a mainstream preschool program for approximately eight hours per day. At the time of the study, he received speech-language therapy once weekly for 30–60 min and had been receiving speech-language services for approximately one year. Prior to this schedule, speech-language therapy was provided on a bi-monthly basis.

Angel, a Native American and Hispanic preschool-aged autistic boy, exposed to English only, had a caregiver-reported spoken vocabulary of more than 200 words. Naomi described Angel as using “gestalts” and reported a history of echolalia, which was confirmed through clinical screening and play observation, during which Angel was observed imitating words and phrases produced by the researcher. While Angel’s expressive communication was not limited to echolalic speech (he had recently developed more flexible, generative language, including 4+ word sentences, early pronouns, concrete verbs, and early descriptors), Naomi reported that Angel continued to rely on rote echolalic phrases, which limited the specificity and overall effectiveness of his communication. Angel reportedly relied on vague vocabulary (i.e., this, that, here, there) and nonspecific phrases, which often resulted in communication breakdowns and frustration when communicating with peers and novel communication partners. His mother also reported that Angel demonstrated difficulty expressing problems, expressing sensory needs, expressing emotions, advocating for his wants/needs, and relaying personal experiences using narratives, further contributing to communication breakdowns. Angel attended a specialized preschool program five days per week. He received physical therapy, occupational therapy, and speech-language therapy services on a weekly/biweekly basis for approximately one year prior to the present study.

#### 2.1.2. Adult Participants

In addition to being a primary caregiver (e.g., parent, grandparent, guardian) for a child who met the inclusion criteria described above, participating caregivers needed to be at least 18 years old, speak English as a primary language, and be available to participate in coaching sessions during the duration of the summer clinic.

Recruitment of student clinicians was completed via convenience sampling of graduate students participating in a summer clinical rotation as part of the Project SCENES interdisciplinary autism training grant program at the University of New Mexico (Gevarter, 2021). It should be noted that participating student clinicians and caregivers were compensated for their time participating in initial and final semi-structured interviews ($25 Amazon gift card codes), and caregivers were compensated for their time participating in caregiver coaching sessions ($10 Amazon gift card codes). Caregivers completed intake forms providing additional information about their and their child’s background, including demographic information, as well as echolalia knowledge and experience. Student clinicians completed intake forms providing additional information about their own demographic background, as well as echolalia knowledge, education and experience. Further information on participant experiences was received via an individual semi-structured interview, conducted with the first author prior to intervention. A summary of adult participant characteristics is provided in Table 2, and an overview of adult participant experience is reported below.

Within Triad 1, Natalie (Remy’s mother) reported not having the opportunity to learn about echolalia but had attended online courses and watched YouTube videos to learn about ASD topics. Emma (Remy’s student clinician) reported falling “in between” feeling “slightly knowledgeable” and “not feel[ing] knowledgeable” about echolalia, stating she has used professional training and self-taught knowledge to learn about echolalia. Emma reported she had taken 1–2 undergraduate or graduate level courses that she recalled discussed echolalia; however, she had not completed any continuing education courses on the topics, nor had any direct interactions with clients who may have used echolalia prior to the study.

Within Triad 2, Naomi (Angel’s mother) described a holistic view of health and development secondary to her work as a nurse and reported prior experience attending a multidisciplinary ASD clinic out of state. The caregiver also noted previous involvement in intervention services, though with limited experience participating in caregiver coaching models. Naomi reported feeling “somewhat knowledgeable” about echolalia, stating she previously heard the term in nursing school to describe language used by a stroke patient. Naomi reported that her overall echolalia knowledge was self-taught, experiential, or based in professional trainings. Liza (Angel’s student clinician) was previously an early childhood educator (for six years), and reported feeling “slightly knowledgeable” about echolalia, learning primarily through self-taught knowledge and experience with a person who uses echolalia. Liza reported she had taken no undergraduate courses, and one graduate-level course that she recalled discussed echolalia. Liza had completed no continuing education courses on the topics and reported she had previous direct interactions with three children who may have used echolalia prior to the study.

### 2.2. Researchers 

The first author acted as the lead clinical instructor for the student clinicians. The lead instructor was a late-diagnosed neurodivergent CCC-SLP and PhD student from a Hispanic background who is a native English speaker and has basic conversational proficiency in French (stronger receptive [listening and reading] than expressive [speaking and writing] skills; able to engage in simple conversations and communicate using everyday vocabulary and basic to advanced sentence structures in familiar contexts). The second author was a first-year graduate student in speech and hearing sciences from a Hispanic background with undergraduate research experience. Author 3 was a certified speech-language pathologist, registered behavior technician, and PhD student. The final, senior author was a BCBA-D and associate professor in speech and hearing sciences who served as the course instructor for a coinciding course in naturalistic intervention.

### 2.3. Setting and Materials

The setting of the research was a university-based speech-language pathology clinic, and research sessions were conducted over a four-week autism summer clinic that involved both group-based therapy (not part of the current study) and pull-out research intervention sessions. Baseline and intervention sessions took place in small therapy rooms (with a child-sized table, chairs, and carpeted play area) with minimal distractions. Based on RAISD preference assessment results, researchers created bags of individualized intervention materials composed of preferred items, toys, and objects. While these play items were not toys owned by the family and were provided by the research lab, researchers aimed to provide play items that were motivating and reinforcing for the child. To reduce material satiation across sessions, researchers created two sets of similar toys (one bag for student clinicians and one bag for caregivers) to be used throughout baseline and intervention. Some larger items such as trampolines remained available across sessions. Example preferred items/activities included a balloon toy, puppets, pretend food, magnetic blocks, and toy cars for Remy and Play-Doh, Mr. Potato Head/Big Feelings Pineapple, cars and ramp, and craft supplies for Angel. Preferred items were placed in the intervention room by research assistants prior to each baseline and/or coaching session. Children were permitted to transition into the research room with preferred or regulating items, as needed. These items remained available throughout the session and, when appropriate, were incorporated into play interactions to follow the child’s interests, support child-led engagement, and maintain positive affective exchanges, consistent with NDBI principles. Researcher assistants utilized timers and audio/video recording devices to record research sessions throughout the study. Communication visual supports were added during coaching sessions. These included an NDBI communication visual outlining NDBI elicitation and response techniques as well as an activity planner adapted from prior studies (Gallegos et al., 2024; McGuire et al., 2025). These materials may be made available upon request.

### 2.4. Research Design

**Figure 1:**
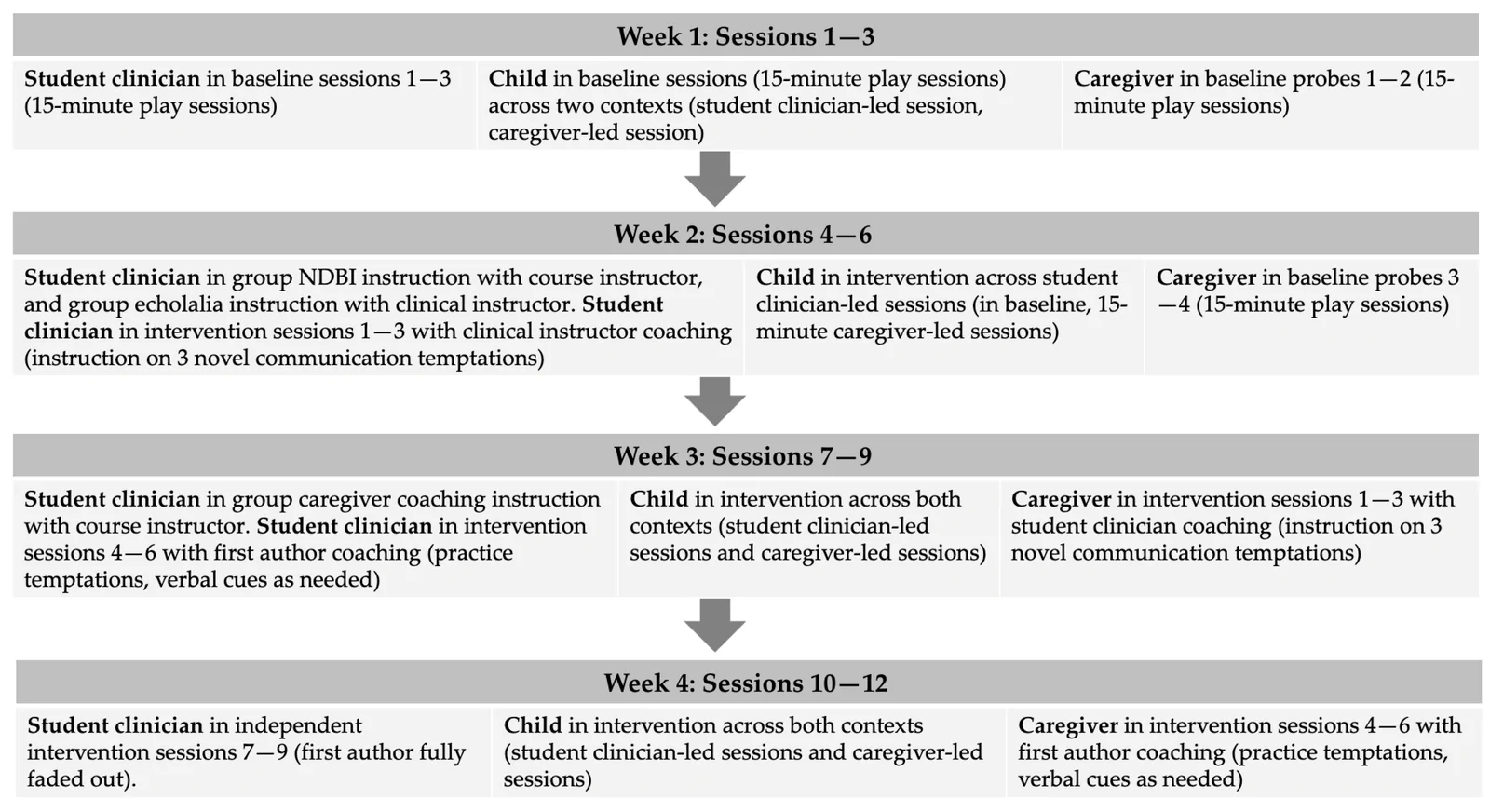
Sequence of Baseline, Intervention, and Coaching Phases Across Student Clinician, Child, and Caregiver Participants.

The present study involved two concurrent implementations of single-case experimental designs: (a) multiple probes across adult participants and (b) multiple probes across contexts (communication partners) that were conducted over the course of four weeks (Gast et al., 2014). Figure 1 provides an overview of the research design and timeline illustrating the sequential introduction of intervention and coaching procedures across student clinician, child, and caregiver participants. Due to duration limitations of the grant-funded summer clinic, the number of baseline probes for adult participants was predetermined. A multiple-probe design was used to reduce repeated exposure to the materials and activities and mitigate participant satiation. During Week 1, three baseline probes were conducted during play sessions in which the student clinician acted as the child’s communication partner (student-led sessions) and two probes were conducted when the parent was the communication partner (caregiver-led sessions). In Week 2, caregivers remained in baseline. So long as they demonstrated low and stable baselines, student clinicians moved to the intervention phase. Specifically, after student clinicians participated in group-based instruction in NDBI and echolalia at the start of Week 2, the lead clinical instructor began coaching students in NDBI techniques during student-led sessions. If student clinicians demonstrated an increase in targeted elicitation and response techniques for at least two sessions in Week 2 (and caregiver-led sessions maintained low and stable baselines), then students began coaching parents during caregiver-led sessions (following group training in coaching) in Week 3. Student clinicians continued to receive coaching support (with gradual fading) from the lead instructor during student-led sessions in Week 3. In Week 4, student clinicians continued to coach caregivers (with gradual support fading if appropriate) and participated in their own sessions with the child independently (no support from the lead instructor). This same design timeline was used to collect data for child participants, who received staggered intervention across contexts (i.e., when interacting with the student clinician or when interacting with their parent).

The study also incorporated elements of a Type I hybrid implementation science design. Semi-structured individual and focus group interviews with student clinicians and caregivers were conducted before and after the intervention, alongside a midpoint survey, to gather participant feedback and inform ongoing refinement of the intervention and coaching procedures. These qualitative data were collected for a separate analysis and are not reported in the present study; implementation-focused findings (e.g., social validity, acceptability, appropriateness, etc.) will be presented in future publications. All study procedures were reviewed and approved by the University of New Mexico Institutional Review Board (IRB). Written informed consent was obtained from all adult participants (student clinicians andcaregivers) prior to participation. Caregivers additionally provided parental consent for their child’s participation, including information about the potential risks and benefits of participation and considerations related to child assent.

### 2.5. Adult Dependent Variables, Operational Definitions and Target Selection

The adult dependent variables within the study included rate of use of targeted (a) NDBI elicitation techniques (create and wait/prompt), and (b) NDBI response techniques (responsive actions and responsive language models).

#### 2.5.1. NDBI Elicitation Techniques

*NDBI elicitation techniques* were operationally defined as adult behaviors designed to evoke a targeted communicative response, including using a targeted communication temptation, providing a wait time before prompting, and delivering a prompt when necessary. Elicitation techniques were grouped into two categories: (a) using target communication temptations (create) and (b) wait/prompt techniques. Separating these components allowed researchers to determine whether adults were successfully following up with additional elicitation methods after initially creating communication temptations.

*Communication temptations* were operationally defined as an adult-arranged manipulation of environment, materials, activity, or interaction to create an opportunity for the child to communicate. Examples included: (a) providing only a portion of a desired item while withholding the remaining pieces (inadequate portions); (b) establishing a familiar routine and pausing to create an opportunity for the child to communicate before continuing the routine (interrupted routine); (c) creating a playful obstacle or unexpected event through actions or language (silly situations) and (d) waiting for the child to request assistance before helping operate a toy or complete an activity (Ousley et al., 2024).

Following baseline sessions, researchers reviewed recorded sessions to identify potential target communication temptations for each adult participant. These were selected from a list adapted from McGuire et al. (2025). Communication temptations aligned with the child’s interests and ongoing activities were targeted if participants demonstrated low rates of responding (≤3 occurrences) with stable performance across at least two baseline sessions. Low-frequency temptations were prioritized to promote the acquisition of new techniques amongst clinicians and caregivers and to expand the range of child communicative forms and functions. Table 3 presents the targeted communication temptations along with examples of individualized intervention activities and routines.

*Wait time* was defined as the adult communication partner pausing for a minimum of 3 s following the creation of a communication temptation. In view of the prevalence of prompt dependence in autistic children (Gorgan & Kodak, 2019), researchers aimed to provide adequate wait time to support initiation and independence (Ousley et al., 2024). *Prompts* were defined as supports to encourage the child’s use of target spoken language communication (Ousley et al., 2024). Prompt types were selected and individualized based on each child’s needs. For Remy, trained prompts included: indirect verbal cues (e.g., *hmm, woah*, *gasp*, *‘what do we do next?’ ‘uh oh’*), non-verbal gestures (e.g., shifting eye gaze, shoulder shrug, thinking face), partial direct verbal models (e.g., /l/ to cue *‘let’s dance,’* single word cue, two-three-word carrier phrase), or full verbal model (e.g., *‘Let’s dance!’ ‘Can I have the [color] [object]?’ ‘I don’t want the [object]’)*. For Angel, trained prompts included: indirect verbal cues (e.g., *hmm, woah, gasp, ‘what next?’ ‘uh oh ‘oh no’*) or gestures (e.g., shifting eye gaze, shoulder shrug, thinking face), partial direct verbal model (e.g., leading question, carrier phrase, single word cue), or full verbal model (e.g., *‘I have a problem.’ ‘The [object]’s not working.’ ‘That’s not a [object], that’s a [object].’*).

#### 2.5.2. NDBI Response Techniques

*NDBI response techniques* (or responsive interactions) were operationally defined as adult behaviors occurring following a child’s communicative act that were intended to reinforce the child’s communication attempt, model new language, or promote engagement with materials. The response techniques were further grouped as *responsive actions* and *responsive language models*. This distinction was informed by previous iterations of the intervention, which suggested adult communication partners often had the most difficulty with techniques involving language models (McGuire et al., 2025; Nattress et al., in press). As a result, response techniques were analyzed separately to examine potential differences in the implementation of action-based and language-based response techniques.

*Responsive actions* included (a) providing natural consequences that matched the child’s communicative act (e.g., providing a requested item, continuing an activity, or assisting with a task) and (b) engaging the child in play after a temptation.

*Responsive language models* included (a) verbally responding to the child’s communicative act through repetition, extensions (adding semantic information), expansions (adding grammatical information), or substitutions that replaced the child’s response with a more contextually appropriate target phrase as well as (b) verbally narrating, describing, or labeling the child’s actions and/or internal states during play.

### 2.6. Child Participant Dependent Variables, Operational Definitions and Target Selection

Child dependent variables included (a) the total number of targeted communication responses (prompted and unprompted) and (b) the total number of unprompted targeted communication responses. *Total* targeted spoken language communication responses were defined as the number of prompted and unprompted child spoken responses following an adult targeted temptation that aligned with the individualized communication targets. *Prompted* targeted spoken language communicative responses occurred following the delivery of one or more prompts by the adult communication partner, regardless of whether the prompt was trained by the coach (e.g., open-ended questions, carrier phrases, full models) or untrained (e.g., yes/no questions). *Unprompted* targeted spoken language communicative responses were produced independently by the child and subsequently reinforced by the adult communication partner following a targeted temptation, without the use of prompts (trained or untrained).

Selection of target child spoken-language communication responses was informed by caregiver interviews, a caregiver echolalia checklist, and researcher observations from screening and baseline sessions. For example, Remy frequently used rote two- to three-word phrases to request objects using nonspecific vocabulary (e.g., “I want this”), whereas Angel commonly used rote two- to four-word phrases to protest or negate using similarly nonspecific language. Information gathered across these sources was integrated to identify developmentally appropriate next steps that would extend each child’s form (syntax), content (vocabulary), and communicative function (verbal operant).

Consistent with the NDBI principle of loose shaping (Schreibman et al., 2015), communication partners were coached to reinforce meaningful approximations of target responses and to gradually support growth across form, content, and use. Specifically, communication partners targeted: (a) utterances of similar length that incorporated more specific vocabulary; (b) utterances of greater length and syntactic complexity, even when vocabulary remained less specific and/or (c) use of existing language forms to communicate novel communicative functions. Given the developmental and individualized nature of these targets, responses were scored as accurate when they demonstrated meaningful growth in at least two of the three dimensions (form, content, or use). Although the specific scoring system employed in this study was developed to evaluate individualized intervention outcomes, the broader framework reflects an established and widely used clinical approach to language assessment (Wilder & Redmond, 2024). Thus, this criterion was selected to capture clinically meaningful approximations of generative communication while remaining sensitive to incremental language growth. Further individualized scoring criteria for each child are described below and summarized in Table 4.

For Remy, whose targets focused on early vocabulary, descriptors, and emerging multi-word combinations, responses were scored as correct when they demonstrated any two-component combination, including (a) form and content, (b) content and communication function, or (c) form and communication function. Given Remy’s early stage of language development, semantically related substitutions (i.e., specific but inaccurate vocabulary) were accepted as demonstrating content. Responses containing only a single component (e.g., nonspecific vocabulary, gestures, vocalizations, or isolated single words) were not considered correct. For example, Remy’s response “I want blue triangle” demonstrated form, content, and use, whereas a semantically related approximation (e.g., “blue square” for a rectangle) was still accepted as demonstrating content in combination with form and use. Inaccurate responses for Remy included productions consisting of only a single component, such as nonspecific single-word utterances (e.g., “this” or “that”), gesture-only responses without verbal output, or nonverbal vocal behaviors (e.g., yelling or screaming), all of which lacked sufficient linguistic form and/or semantic content to meet criteria for accuracy.

For Angel, whose targets included more advanced linguistic concepts such as multi-word utterances, complex sentence structures, and precise vocabulary, responses were scored as correct when they demonstrated any two-component combination, including (a) form and content, (b) content and use, or (c) form and use. In addition, accurate responding required the use of precise vocabulary (i.e., specific and accurate vocabulary terms). For Angel, responses containing only a single component, even if precise and accurate, were not considered correct. For example, Angel’s response “That’s not a nose, that’s teeth” demonstrated form, content, and use, whereas “That’s not right” demonstrated form and use and still met criteria for accuracy under the loose shaping framework. Responses containing only a single component, even when characterized by precise and accurate vocabulary, were not considered correct. For example, Angel’s response “help,” produced without additional linguistic information (e.g., “Help me fix it,” “fix the car”), reflected communicative intent but lacked sufficient structural complexity and semantic specificity, and therefore, did not meet criteria for accuracy.

### 2.7. Procedures for Student Clinician Baseline, Student Clinician Group Instruction on NDBI Techniques, and Instructor-Led Coaching Sessions for Student Clinicians

#### 2.7.1. Baseline (Week 1)

During the larger group clinic time, child participants and their supporting student clinician were pulled for 15 min daily to conduct baseline probes in a separate room where the child’s play materials were available. Three baseline probes were conducted (one per day). Caregivers were not present, and the student clinicians were instructed to interact and play with the child as they typically would. The observation began once the child demonstrated interest in or initiated engagement with an item or activity. The lead clinical instructor observed sessions but did not provide coaching or feedback.

#### 2.7.2. Student Group Instruction in NDBI and Echolalia (Week 2)

In Week 2, student clinicians participated in large group NDBI instruction and small group echolalia instruction (each approximately two hours in length). Group NDBI instruction (occurring the day before coaching began) was embedded within a university course that included the two participating student clinicians as well as five other students. Before the in-person course, students completed an online Naturalistic Intervention module (Amsbary & AFIRM Team, 2025a) and readings on goal development and communication (Minjarez et al., 2020a; Bruinsma et al., 2020). Rather than teaching a specific manualized NDBI approach, instruction emphasized core NDBI techniques common across models. The course instructor used behavioral skills training (BST; Miltenberger, 2011) to teach, model, and practice key NDBI communication techniques. Specifically, students learned NDBI elicitation techniques (using communication temptations, waiting, prompting) and NDBI response techniques (natural reinforcement, language modeling, and allowing interaction time), with added emphasis on maintaining play engagement and following the child’s lead. Training included visual aids, in situ models, video models (from prior studies), small group roleplay, and feedback.

Immediately following group NDBI instruction, the lead clinical instructor provided targeted training on echolalia to the two participating student clinicians. Instruction adopted a strengths-based, neurodiversity-affirming framework, conceptualizing echolalia as functional (e.g., communicative, regulatory, learning; Cohn et al., 2024; Dinello & Gladfelter, 2025) and a stepping stone towards generative language (Cohn et al., 2023b). Students were trained to identify different types of echolalia (immediate echolalia, delayed echolalia; Lord et al., 2012), hypothesize communicative intent using contextual cues and child history, and select developmentally appropriate targets that expand language form, content, and use. Emphasis was placed on acknowledging and building upon echolalia through contingent responding, loose shaping and modeling language at the child’s level plus one, while supporting flexible, functional communication and engagement.

#### 2.7.3. Clinical Instructor-Led Coaching and Fading for Student Clinicians (Weeks 2–4)

Following group instruction, student clinicians participated in 30–40-min coaching sessions that included a 15-min play interaction. A coaching checklist supported coaching fidelity throughout week two coaching sessions. During the first 10–15 min of the coaching session, the clinical instructor used strategy instruction and the NDBI communication visual support to review NDBI elicitation and response techniques. The clinical instructor and student clinician then jointly completed the activity planner by identifying: (a) a target temptation; (b) motivating, play-based activities; (c) wait time; (d) selected prompting techniques; (e) target child responses; (f) responsive actions based on the child’s communication responses and subsequent play behaviors (natural consequences matched to communication/play engagement) and (g) responsive language models aligned with the child’s communication responses and subsequent play behaviors (mapped to the child’s communicative act ). While filling out the planner, the instructor provided guidance on targeted child communication responses for echolalic language, as well as echolalia-specific adaptations to prompting techniques and response techniques. In view of pronoun use patterns by some children who use echolalia (substituting “you” for “I” pronouns), student clinicians were instructed to use personal pronouns from the child’s perspective within prompts and responsive language models to facilitate accurate pronoun use, should the child immediately repeat the prompt/model (immediate echolalia). For example, for Remy, who frequently imitated verbal language models such as “Do you want to jump?” adult participants were coached to prompt using “I” phrases rather than questions, such as “I want to jump,” which were then imitated by the child, and reinforced by the adult. Additionally, students were advised to use loose shaping to reinforce verbal communication attempts that represented incremental advances (“one step above”) the child’s current abilities in syntax/grammar, content (e.g., vocabulary), and/or use (e.g., pragmatic functions). Similarly for Remy, adult participants were instructed to reinforce approximations of target responses; when Remy incorrectly verbalized “Let’s bounce” to direct the adult to kick the ball within a turn-taking routine, the adult provided a contingent verbal response of the target “Let’s kick” and then reinforced by kicking the ball, continuing the routine. Although the clinical instructor identified three levels of prompting intensity along a least-to-most continuum (e.g., exaggerated facial expression or verbal cue “oh no” --> carrier phrase “I have…” --> full model “I have a problem”), strict adherence to a prompting hierarchy was not required to minimize prompt dependence and increase independent use of skills (Gorgan & Kodak, 2019). Instead, student clinicians were encouraged to implement varied prompts responsively, based on the child’s immediate needs. After sequentially planning and discussing each component, the clinical instructor provided direct models of technique use with the child.

Following the explicit instruction, student clinicians then engaged the child in a 15-min play interaction. During these interactions, the instructor provided in-the-moment coaching through verbal feedback, assistance, and modeling as needed. This also included verbal suggestions and models for interspersing easier tasks with more challenging targets in response to observable indicators of child dysregulation or frustration to maintain motivation, assent, and positive engagement. Examples of observable behaviors that indicated difficulty with regulation included: running, jumping, crashing into objects, throwing materials, pushing objects away, yelling “no,” dropping to the floor, eloping from the activity area, or withdrawing from interaction. After the play interactions, the clinical instructor answered relevant questions and provided specific performance feedback on a minimum of three NDBI techniques.

In Week 3, no new targets were added, with sessions focusing on review, practice, and feedback on the use of the existing three communication temptations and corresponding NDBI techniques. The NDBI communication visual support and the collaboratively completed activity planner developed during the first week were available, and the lead clinical instructor continued coaching students during 15-min play interaction sessions, gradually reducing support as appropriate. In Week 4, the student clinician independently implemented NDBI intervention sessions with their child participants without coaching support from the clinical instructor (not present).

### 2.8. Procedures for Caregiver Baseline, Student Clinician Group Instruction in Coaching, and Student Clinician-Led Coaching Sessions for Caregivers

#### 2.8.1. Baseline (Weeks 1 and 2)

Caregiver sessions occurred 30 min prior to the start of the group clinic time in the small therapy room. Four baseline probes were staggered across weeks one and two (across six days) to prevent satiation with materials. The caregivers, child, and student clinicians were present, but the clinical instructor was not available. Child play materials were available, and the student clinician instructed the caregiver to interact and play with their child as they typically would. The observation began once the child demonstrated interest in or initiated engagement with an item or activity. The student clinician did not provide any assistance or feedback.

#### 2.8.2. Student Clinician Group Instruction in Coaching (Week 3)

During group caregiver coaching instruction, student clinicians received two hours of group instruction on caregiver coaching within the university course. Students completed an online parent-mediated intervention module and assigned readings on parent coaching (Amsbary & AFIRM Team, 2025b; Minjarez et al., 2020b). Instruction during the first hour included general family-centered practices and, during the final hour, BST was used to teach the specific coaching procedures used in this study. These included joint planning (via activity planner or brief check-ins), strategy instruction (describing and modeling techniques with the aid of visual supports), guided caregiver–child play with in-the-moment feedback, and post-session feedback targeting key communication techniques. The instructor described and demonstrated each step (using in-situ and video models), provided coaching checklists, and facilitated role-play practice with feedback (Gevarter et al., 2021).

#### 2.8.3. Student Clinician-Led Coaching for Caregivers (Weeks 3 and 4)

As both student clinicians demonstrated increased use of targeted NDBI techniques across at least two sessions during week two, they began leading caregiver coaching sessions during week three (the day after group instruction in coaching). Student clinicians were provided with coaching checklists to support fidelity of coaching implementation (Gallegos et al., 2024; McGuire et al., 2025). Students then followed the same procedures for coaching that were modeled for them during week 2 by the clinical instructor (i.e., introducing one temptation daily via strategy instruction, jointly completing the activity planner, providing guidance and assistance during play interaction, and giving explicit feedback on at least three NDBI techniques). It should be noted that, as appropriate, Emma spoke to Natalie in Dutch/German within sessions to build rapport; however, coaching was primarily conducted in English.

In week 4, student clinicians continued to coach caregivers using the faded support approach modeled to them by the clinical instructor in week 3. This meant no new targets were added, but students reviewed visuals, answered questions, and gradually reduced support as appropriate during the play interactions.

### 2.9. Data Collection 

#### 2.9.1. Coding of Adult Dependent Variables

All 15 min play sessions were video recorded, and coders used a standardized data sheet to document adult implementation of seven observable behaviors: (a) using a target communication temptation (create); (b) pausing for at least 3s before prompting (wait); (c) delivering a prompt when necessary; (d) responding to the child’s communicative behavior with a natural consequence matched to the communicative act (responsive actions); (e) repeating, expanding, extending or substituting the child’s communicative act (responsive language models); (f) providing opportunities for continued engagement with materials or activities (responsive actions) and (g) verbally describing the child’s play or actions (responsive language models).

*Create* was scored as correct, with timestamp noted, when the adult participant was observed creating a target communication attempt. If the coach (clinical instructor or student clinician) created the communication temptation alone, the communication temptation was not coded. If the coach modeled creation of a temptation that the adult then imitated, create was scored as correct. *Wait* was scored as correct when the adult provided a minimum of 3s of wait time. *Prompt* was scored as correct when the adult delivered the specified prompt intended to evoke the target child communication response. *Responsive actions* were scored as correct when the adult provided both natural consequences matched to the child’s communication and opportunities for continued engagement with materials or activities. *Responsive language models* were scored as correct when the adult used a language model after the child’s communicative act and verbally described the child’s play or actions. Consistent with child-led NDBI principles, elicitation and response technique components were coded as not applicable (NA) when the child’s independent behavior rendered implementation unnecessary. For example, elicitation techniques (wait, prompt) were coded as NA when the child independently produced a targeted communicative response following a communication temptation before prompting was required. Additionally, response techniques were coded as NA when the child independently maintained engagement and/or immediately transitioned into the next communication temptation without engaging in play due to increased motivation. NA opportunities were treated as correct implementations during scoring because withholding reinforcement or unnecessarily prompting would have been inconsistent with responsive, child-led interaction.

#### 2.9.2. Coding of Child Dependent Variables

For child target communication outcomes, the frequency of total and unprompted target communicative responses was recorded using the same data system employed for adult behaviors. Based on operational definitions, coders classified the communicative response following each targeted temptation as either prompted or unprompted. Importantly, only one target child spoken language response was scored per targeted temptation (the last response made during a turn). For example, if a child produced a non-target response (e.g., less advanced verbal response, “more bubbles”) and the communication partner subsequently prompted a target response (e.g., “I want to blow bubbles,” “Let’s blow bubbles”), which the child then produced, the response was coded as a prompted target. If there were instances in which the partner accepted/reinforced a non-verbal response (e.g., rocking body forward in response to interrupting routine) or a spoken language response that did not meet operational definitions (e.g., yelling or shouting ‘no’ in response to communication temptation), these were considered non-targets and coded as incorrect. The total number of target communicative responses per session was calculated by summing all prompted and unprompted responses that received reinforcement in relation to adult-delivered targeted temptations. These totals were then converted to rates per minute by dividing the number of responses by the 15-min session duration. Rates of total and unprompted target communicative responses were subsequently graphed across baseline and intervention phases for visual analysis.

#### 2.9.3. Coding Interobserver Agreement (IOA) 

The first author served as the primary coder for both triads across adult and child dependent variables. For each adult and child participant, 33% of sessions were randomly selected for inter-observer agreement (IOA). The independent observer for Remy’s sessions was the third author, and the independent observer for Angel’s sessions was the second author. The first author independently trained both independent observers using 10% of sessions across both triads using a researcher-developed data sheet. Average IOA for adult dependent variables was 89.5% (range of 85.7–97.7%) and 100% (all sessions 100%) for Triad 1 student-led and caregiver-led sessions, respectively, and 88.3% (range of 67.0–100%) and 84.8% (range of 75.0–100%) for Triad 2 student-led and caregiver-led sessions, respectively. The observed variability in IOA was impacted by sessions in which there was an overall low number of opportunities or the need to continue to refine and norm on operational definitions for response strategies. Discrepancies were discussed, and consensus was reached.

Child data were initially coded alongside the adult dependent variables using the same data sheet used for coding adult dependent variables. Upon initial review of this data, it was determined that operational definitions for child outcomes needed to be further refined (e.g., to clearly distinguish between prompted and independent responses, and vague versus specific vocabulary).

For Remy’s sessions, the first and third authors collaboratively coded three videos together (13.6% of 22 sessions) to build consensus on revised operational definitions. For Angel’s sessions, the first and second authors then collaboratively coded two videos together (9.1% of 22 sessions) to build consensus on refined operational definitions. All child data were then re-coded using a data sheet specifically designed to provide more detailed coding instructions. Average IOA for child dependent variables for Triad 1 (Remy) was 96.1% (range of 76.9–100%) for total communication responses and 88.3% (range of 60–100%) for unprompted communication responses. Average IOA for child dependent variables for Triad 2 (Angel) was 91.3% (range of 60–100%) for total communication responses and 83.5% (range of 50–100%) for unprompted communication responses. The observed variability in child-dependent variables was largely attributable to discrepancies in the identification of prompted responses. The secondary coder was a master’s level student, and discrepancies in applying the operational definitions were identified during coding. The operational definitions were subsequently reviewed and clarified, and an additional PhD-level student was selected to complete the remaining coding. Discrepancies were discussed among coders, and consensus was reached through discussion.

### 2.10. Treatment Fidelity

Group instruction fidelity was supported using researcher-generated BST checklists. The first author independently reviewed sessions using checklists and confirmed 100% fidelity across both week two and week three group trainings. Coaching fidelity was assessed for 33% of intervention sessions using video recordings collected for IOA. The first and third authors used coaching checklists to document implementation of coaching steps by both the lead instructor and student clinicians. The independent observer for all treatment fidelity sessions was an undergraduate research assistant, who utilized the coaching checklist as a fidelity guide. Average coaching fidelity for the lead instructor was 100% for Triad 1 and 100% for Triad 2. Student clinician fidelity averaged 90% for Emma (Triad 1) and 100% for Liza (Triad 2).

### 2.11. Data Analysis 

For adult participant outcomes (student clinician and caregiver), the two primary dependent variables were graphed across baseline and intervention phases. Data were analyzed using visual analysis procedures (Kratochwill et al., 2013), including evaluation of level, trend, variability, and immediacy of effect. For child participant outcomes, the two primary dependent variables were graphed across baseline and intervention phases, and analyzed using visual analysis procedures (Kratochwill et al., 2013), including evaluation of level, trend, variability, and immediacy of effect. Due to the short-term nature of the study and the anticipated variability in responding in natural environments, a mastery criterion was not set.

Tau-U was also calculated as a nonoverlap effect size metric that complements visual analysis, controls for baseline trend, and incorporates both level and trend changes (Brossart et al., 2014; Rakap, 2015). Tau-U values were generated usingthe Single Case Research web-based calculator (Version 2.0; Texas A&M University, College Station, TX, USA; Vannest et al., 2016)with 90% confidence intervals. Effect sizes were interpreted based on established guidelines (Vannest & Ninci, 2015): values ≤ 0.20 indicated small effects, 0.21–0.60 moderate effects, 0.61–0.80 large effects, and ≥0.81 very large effects.

## 3. Results

The results of the study are presented for each participant and organized by participant type (adult and child). Adult participant results are presented first to evaluate changes in the implementation of targeted NDBI techniques across phases. Child participant results are then presented to examine changes in communication outcomes across communication contexts.

### 3.1. Adult Participant Effect Sizes

Across both adult participants in Triad 2, data revealed very large effect sizes for all dependent variables, while Triad 1 was more variable. Specifically, in Triad 1, Emma (student clinician) had very large effect sizes for all dependent variables except for responsive language models, which had large effects. Natalie (Triad 1 caregiver) had large effects for all elicitation techniques, and moderate effects for all response techniques. Table 5 presents Tau-U effect sizes and corresponding *p*-values for student clinician and caregiver implementation of NDBI techniques.

### 3.2. Adult Participant Visual Analysis

Visual analysis results for the adult participants are presented by triad. Visual analysis suggested a functional relationship between the NDBI cascading coaching model and adult dependent variables are present for both adults in Triad 2, as well as the student clinician in Triad 1. Visual analysis results for the Triad 1 caregiver (Natalie) are more mixed.

#### 3.2.1. Adult Participant NDBI Elicitation Techniques

**Figure 2:**
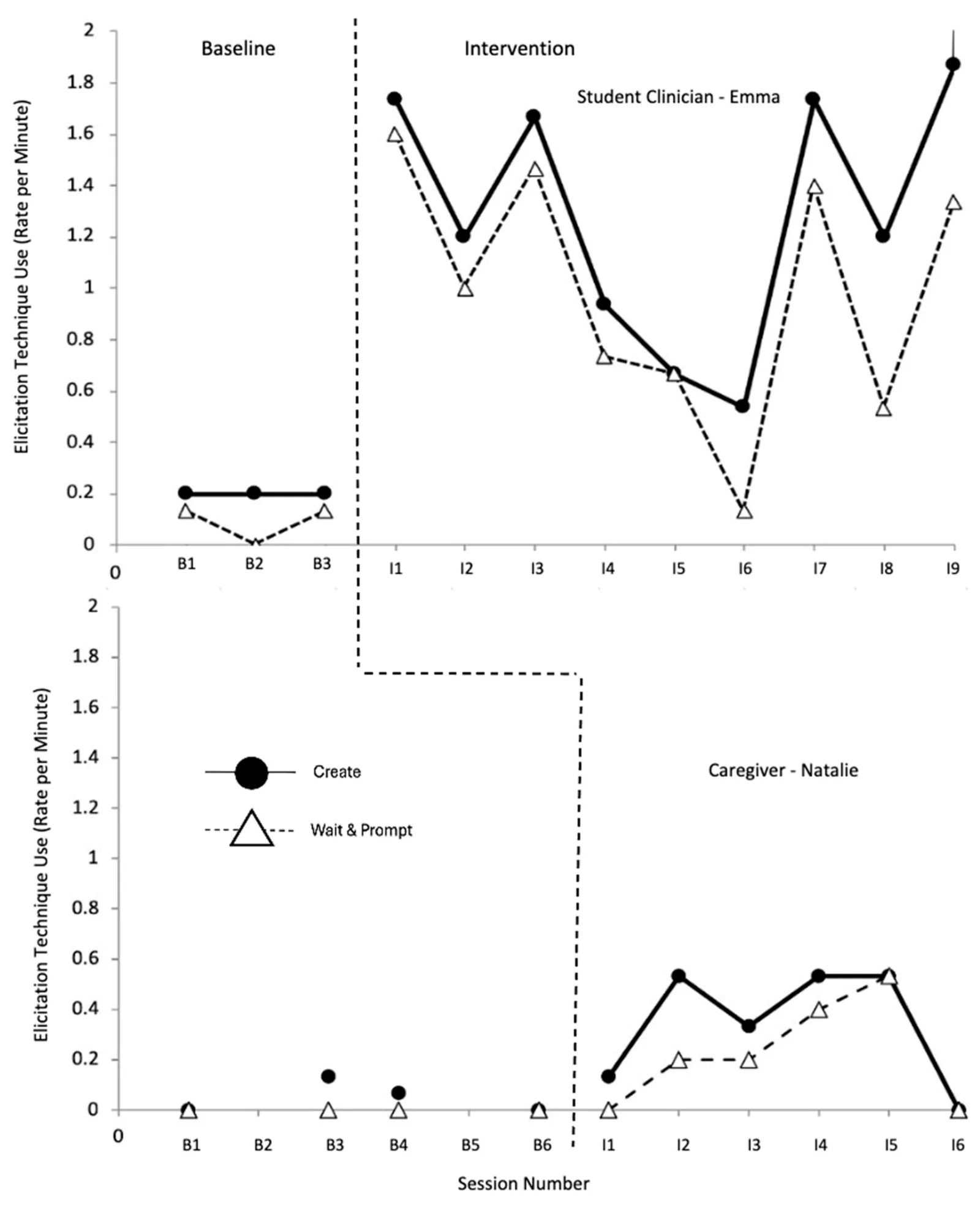
Triad 1 student clinician, and caregiver rate of targeted *elicitation* techniques per minute during a 15-min play session. **Note.** Data points to the left of the vertical dotted line represent the baseline phase, whereas data points to the right represent the intervention phase. The vertical dotted line indicates the phase transition.

Data for Triad 1 elicitation techniques are displayed in Figure 2. Emma (student clinician) maintained low, relatively stable baselines for both creating communication temptations and wait/prompt techniques. There was a clear and immediate increase in her use of elicitation techniques once coaching began. Although there was high variability, she had an average rate of 1.28/min for creating temptations (approximately one opportunity every 0.8 min) and a 0.99/min rate for wait/prompt (approximately every 1.01 min). Data trends were relatively neutral, with decreases noted in intervention sessions four through six (week of faded coaching). However, data recovered to near-maximum levels within sessions seven and nine when the coach was not present. Throughout all intervention and independent sessions, Emma’s use of “wait and prompt” elicitation techniques increased yet remained slightly below her creation of targeted communication temptations throughout intervention.

It is important to note that during intervention sessions four through six, Remy demonstrated multiple instances of visible dysregulation (e.g., running, crashing, throwing objects, screaming). During session five specifically, Emma reported Remy was in a “heightened emotional state” with “a lot of screaming” and “big refusals.” The decrease in elicitation strategy use during these sessions overlapped with the initiation of same-day caregiver intervention sessions, requiring Remy to participate in two coaching sessions per day (compared with two baseline sessions, or one baseline and one intervention session previously).

In baseline sessions, Natalie showed low, stable levels of communication temptations and wait/prompt techniques. While a clear immediacy of effect was not evident, Natalie demonstrated gradual increases in her use of both create and wait/prompt techniques. It should be noted that for caregiver intervention session one, Natalie reported she “stayed up all night” prior to the session, and appeared lethargic (e.g., yawning, briefly sleeping), at which time, Emma joined and engaged with the child. After that first session, there was a change in level followed by a slight positive trend until session six. During this session, Natalie reported Remy felt unwell (i.e., gastrointestinal issues) and, as a result, Natalie engaged playfully with her child, narrating his play; however, she did not create any opportunities for him to use target responses. During intervention, Natalie’s average rate of creating opportunities was 0.33/min for create (approximately one opportunity every 3.03 min) and 0.22/min for wait/prompt (approximately one every 4.5 min).

**Figure 3:**
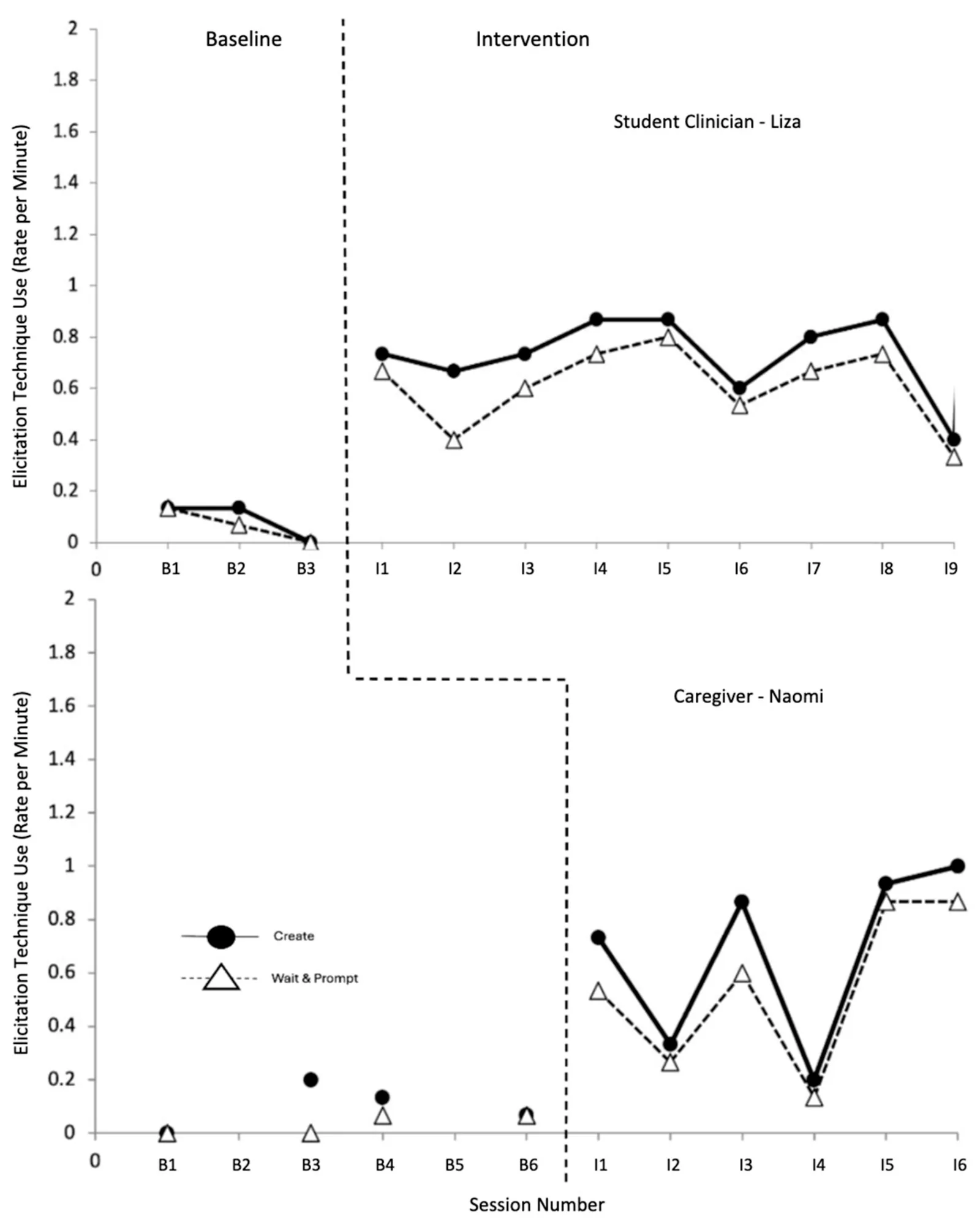
Triad 2 student clinician and caregiver rate of targeted *elicitation* techniques per minute during a 15-min play session. **Note.** Data points to the left of the vertical dotted line represent the baseline phase, whereas data points to the right represent the intervention phase. The vertical dotted line indicates the phase transition.

Data for Triad 2 elicitation techniques is displayed in Figure 3. Throughout baseline sessions, Liza maintained a low, relatively stable baseline for all elicitation techniques. After coaching began, there was a clear and immediate increase in her use of elicitation techniques, which was maintained with some variability. The average rate per minute across intervention was 0.73/min for create (approximately one opportunity every 1.4 min) and 0.61/min for wait/prompt (approximately one every 1.6 min). Data trends were relatively neutral. Throughout intervention, Liza’s use of wait/prompt remained slightly below her use of targeted temptations.

Throughout baseline probes, Naomi maintained a stable baseline of zero targeted elicitation techniques. After coaching began, there was a clear, immediate increase in her use of elicitation techniques. Throughout intervention, Naomi demonstrated high variability of elicitation technique use with average rates per minute of 0.74/min for create (approximately one every 1.4 min) and 0.57 for wait/prompt (approximately one every 1.8 min) across sessions. There was a decrease in her use of elicitation techniques during the second coaching session, when she learned a second new communication temptation, as well as within coaching session four. Overall, a positive trend can be observed in her use of elicitation techniques across coaching sessions. Naomi demonstrated similar levels of create and wait/prompt technique use for half of her sessions, with slightly lower levels of wait/prompt for the other half.

#### 3.2.2. Adult Participant NDBI Response Techniques

**Figure 4:**
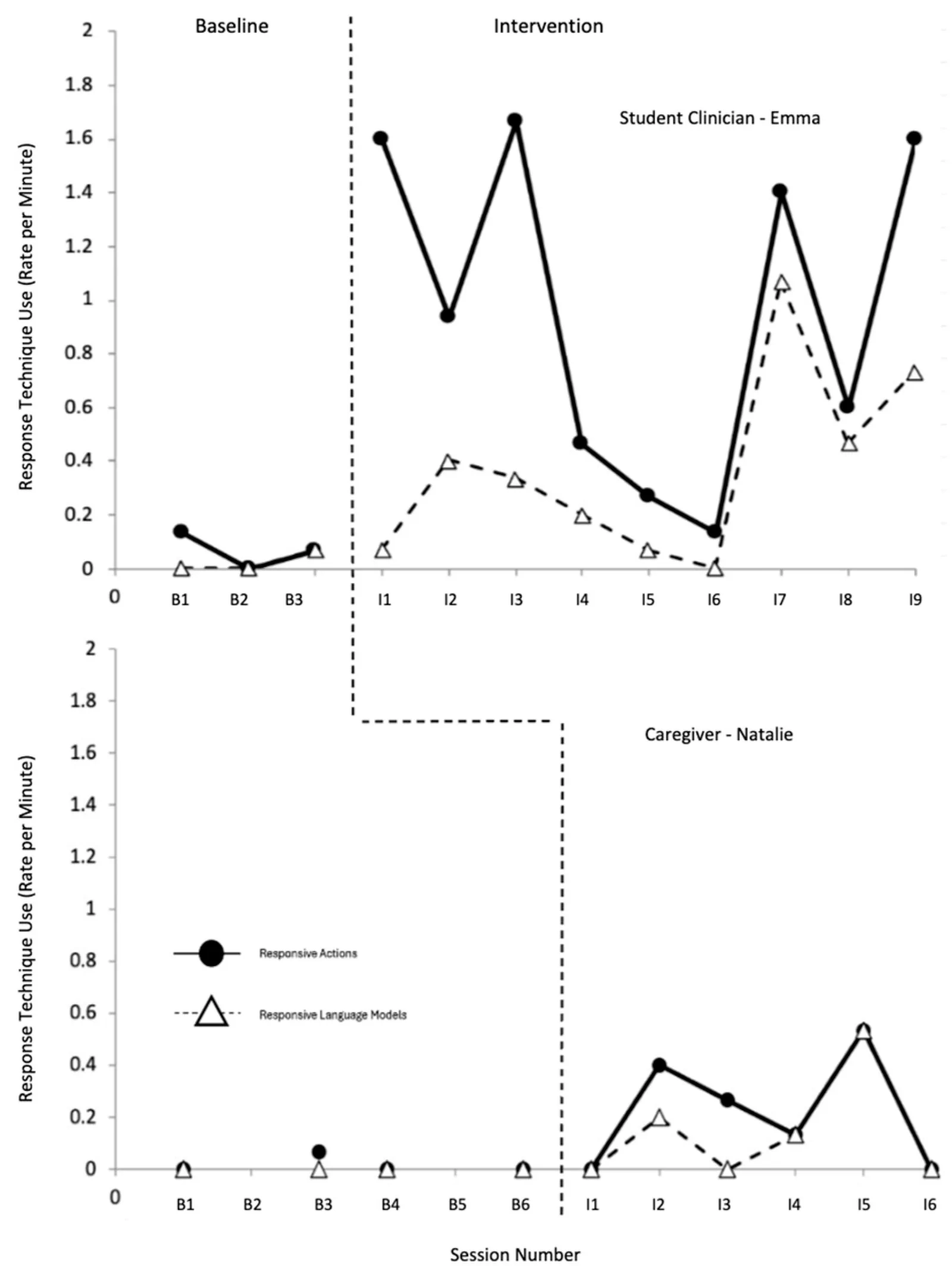
Triad 1 student clinician and caregiver rate of targeted *response* techniques per minute during a 15-min play session. **Note.** Data points to the left of the vertical dotted line represent the baseline phase, whereas data points to the right represent the intervention phase. The vertical dotted line indicates the phase transition.

Data for Triad 1 response techniques are displayed in Figure 4. Throughout baseline, Emma had low, stable levels of response technique use (responsive actions and responsive language models). A clear immediate increase in responsive actions can be seen when coaching began, with a decrease in technique use in intervention sessions four through six, commensurate with levels of elicitation techniques used (see Section 3.2.2 above). Rates of responsive language models gradually increased between intervention sessions one and two, followed by a steady decrease through intervention six. Throughout sessions one through four, Emma had consistently lower rates of responsive language models compared with responsive actions. It should be noted that throughout these sessions, Emma was observed using non-specific verbal praise such as “good job!” or “good talking!” rather than specific verbal language models (e.g., restating target response, expanding, etc.). Following these sessions, rates of both responsive actions and responsive language models rebounded to higher levels and Emma’s use of responsive language models became more comparable to use of responsive actions. Overall, average rates per minute were 0.96/min for responsive actions (approximately one responsive action every 1.0 min) and 0.37/min for responsive language models (approximately one responsive language model every 2.7 min).

Throughout baseline, Natalie maintained a stable baseline level of zero targeted response techniques (responsive actions and responsive language models). In view of the caregiver behaviors within intervention session one (see Section 3.2.2 above), Natalie demonstrated a marginal increase in her use of responsive actions during sessions two to five, with more variability in responsive language models. Overall, data trends were neutral. As she did not create any opportunities for target responses during session six (when she reported Remy was not feeling well), there were no opportunities to use response techniques in this session. Across the intervention, she used responsive actions at a rate of 0.22/min (approximately one every 4.5 min) and responsive language models at a rate of 0.14/min (approximately one every 7 min).

**Figure 5:**
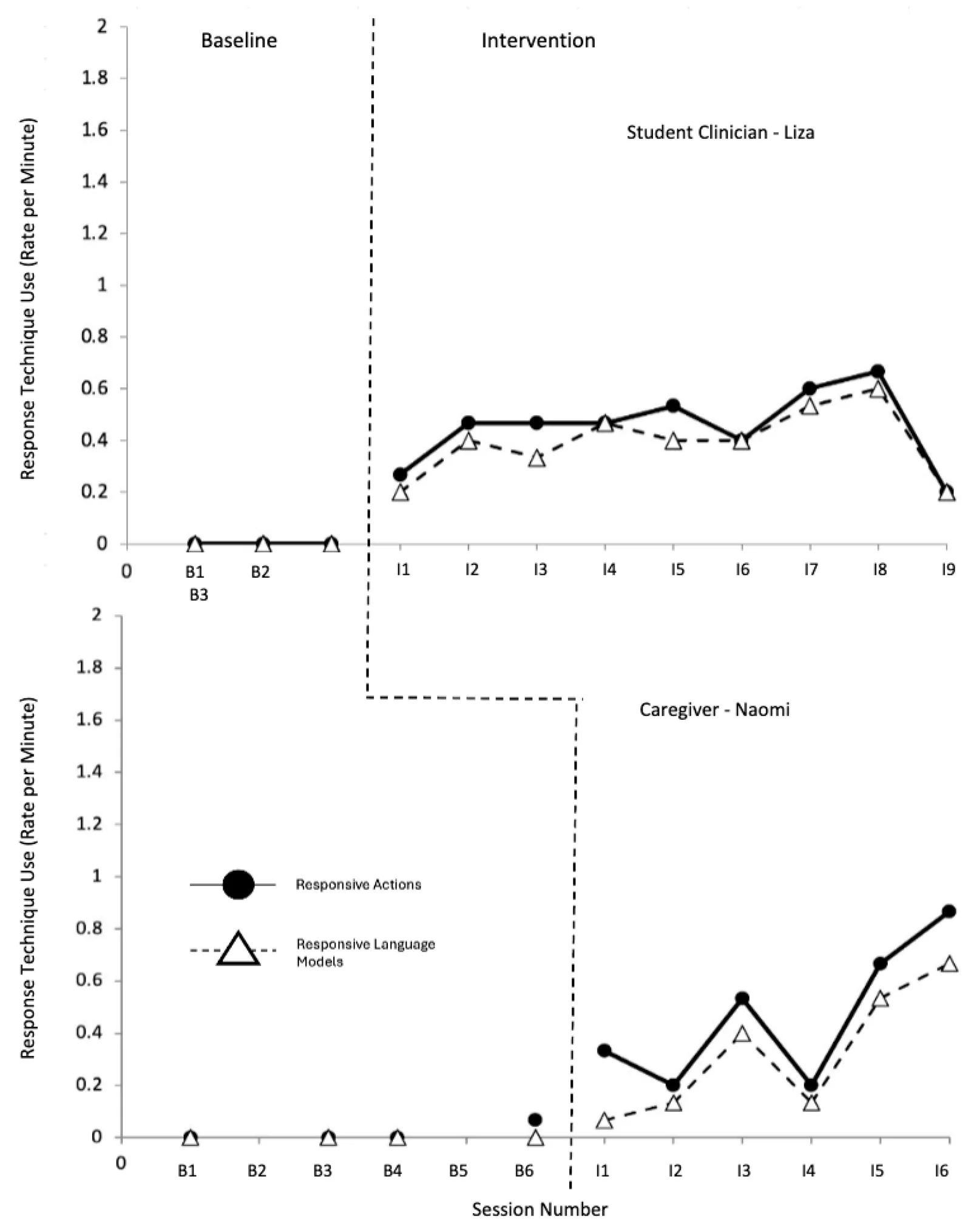
Triad 2 student clinician and caregiver rate of targeted *response* techniques per minute during a 15-min play session. **Note.** Data points to the left of the vertical dotted line represent the baseline phase, whereas data points to the right represent the intervention phase. The vertical dotted line indicates the phase transition.

Data for Triad 2 response techniques are displayed in Figure 5. Throughout baseline, Liza maintained a stable baseline of zero targeted response techniques for both responsive actions and responsive language modeling. Once coaching was introduced, there was an immediate increase in her use of response techniques. The rate of response technique use rose sharply and continued to increase with an overall gradual positive trend before a dip in the last session. Overall, average rates per minute were 0.45/min for responsive actions (approximately one every 2.2 min) and 0.39/min for responsive language models (approximately one every 2.6 min). Liza’s use of responsive actions remained generally higher than her use of responsive language models for most sessions.

Throughout baseline, Naomi maintained a stable baseline of zero targeted responsive actions and responsive language models. There was an immediate increase in response techniques once coaching was introduced (with a larger increase in level of responsive actions). Throughout the intervention, there was moderate variability and a strong positive trend. Overall, average rates per minute were 0.48/min for responsive actions (approximately one every 2.0 min) and 0.32/min for responsive language models (approximately one every 3.1 min). Naomi’s use of responsive actions remained slightly above her use of responsive language models.

### 3.3. Child Participant Effect Sizes

Across student clinician-led sessions, Remy and Angel had very large effect sizes for total (prompted + unprompted) spoken language responses. Remy also had a very large effect size for unprompted responses with his student clinician and Angel had a large effect size. Across caregiver-led sessions, Angel (Triad 2) had a very large effect for total responses and a large effect for unprompted responses. Remy (Triad 1) had moderate effects for both total responses and unprompted responses during his caregiver sessions. Table 6 displays all child Tau-U effect sizes.

### 3.4. Child Targeted Communication Response Visual Analysis

Data for Remy (Triad 1) and Angel (Triad 2) are examined individually. Findings across child participants suggest functional relationships between the NDBI cascading coaching model and total target communication responses across adult partners, with more mixed effects on unprompted target responses (greater changes seen during student clinician sessions).

#### 3.4.1. Remy’s Targeted Spoken Language Communication Responses

**Figure 6:**
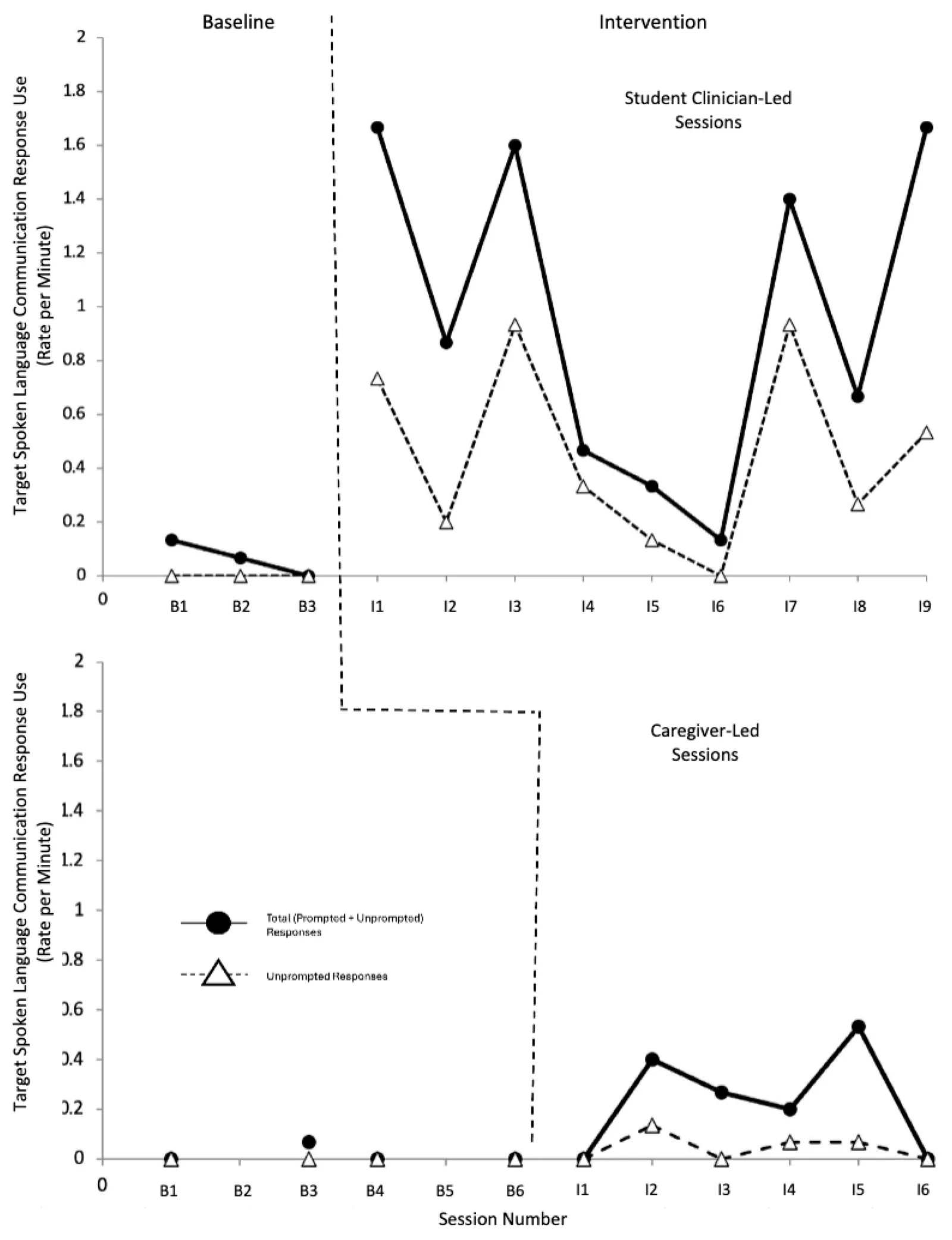
Remy’s rate of total and unprompted targeted spoken language communication responses per minute across 15-min student clinician-led sessions and caregiver-led sessions. **Note.** Data points to the left of the vertical dotted line represent the baseline phase, whereas data points to the right represent the intervention phase. The vertical dotted line indicates the phase transition.

Remy’s targeted spoken language communication response data across contexts (student clinician-led sessions, caregiver-led sessions) is illustrated in Figure 6. Within student clinician-led sessions, Remy demonstrated immediate increases in rates of total targeted (prompted + unprompted) spoken language communication responses with high variability during intervention, reflecting total targeted communication response rates of 0.98/min (corresponding to approximately one response every 1.0 min). While some overlap can be seen with baseline rates, Remy’s use of unprompted response rates immediately increased when intervention began. Although data were highly variable (corresponding to adult participant behaviors), his average unprompted response rate during intervention was 0.45/min (approximately one response every 2.2 min).

Within caregiver-led sessions, there was an immediate increase in his total target response rate followed by an overall neutral trend with minimal variability. His average intervention rate of total targeted responses was 0.23/min, corresponding to approximately one response every 4.3 min. Unprompted responses reflected comparatively lower levels, with significant overlap between baseline sessions. His average intervention rate of independent targeted responses was 0.04 per minute, corresponding to approximately one response less than every 15 min.

#### 3.4.2. Angel’s Targeted Spoken Language Communication Responses

**Figure 7:**
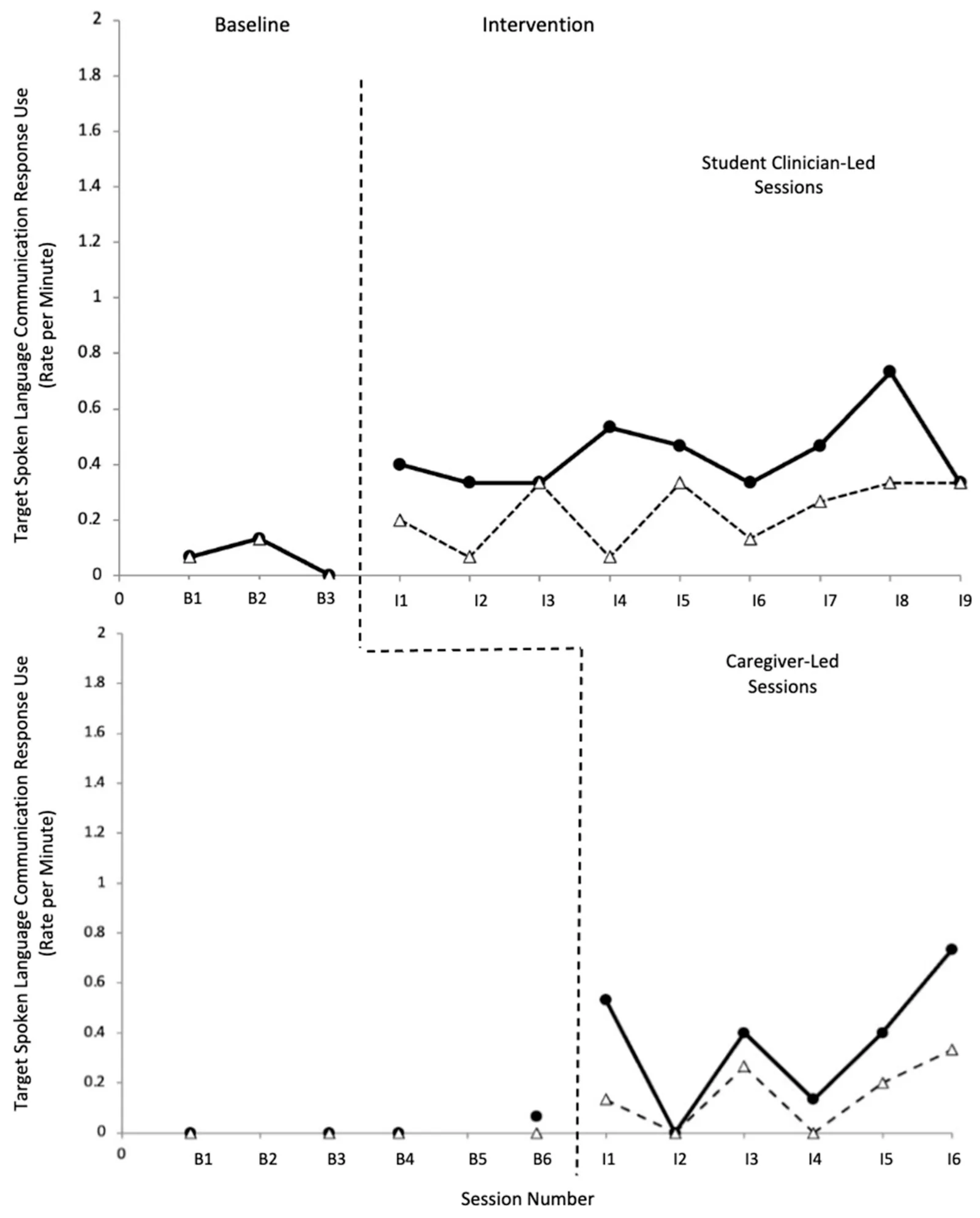
Angel’s rate of total and unprompted targeted spoken language communication responses per minute across 15-min student clinician-led sessions and caregiver-led sessions. **Note.** Data points to the left of the vertical dotted line represent the baseline phase, whereas data points to the right represent the intervention phase. The vertical dotted line indicates the phase transition.

Angel’s spoken language communication response data across contexts is illustrated in Figure 7. Angel demonstrated low, stable levels of targeted communication responses (total and unprompted) within baseline probes across student clinician-led and caregiver-led sessions. Within intervention phases across contexts, there were strong demonstrations of immediacy of effect. Level increases in total responses (average intervention rate of 0.43 min, corresponding to approximately one response every 2.3 min) as well as unprompted responses (average intervention rate of 0.23 per minute, corresponding to approximately one response every 4.4 min) were observed within student clinician-led sessions, with minimal variability and an overall neutral trend.

Within caregiver-led sessions, there were immediate level increases in total responses (average intervention rate of 0.37 per minute, corresponding to approximately one response every 2.7 min) and an overall positive trend. Unprompted responses followed a more gradual increasing trend, with an average intervention rate of 0.16 per minute, corresponding to approximately one response every 6.3 min.

## 4. Discussion

The brief cascading coaching model resulted in an increase in the use of NDBI elicitation and response techniques amongst SLP graduate student clinicians and caregivers of echolalic, autistic children. These findings extend similar results reported in studies examining cascading coaching models for clinicians and caregivers of autistic children with minimal spoken language (Gallegos et al., 2024; Gevarter et al., 2021; McGuire et al., 2025; Nattress et al., in press). Notably, NDBI cascading coaching intervention with echolalia-responsive adaptations was associated with an increase in targeted child spoken communication responses.

It is important to note that visual analysis and effect size findings revealed differentiated patterns across triads and participants. Clear functional relations were observed across both adult and child participant dependent variables in Triad 2 (with all effects being large to very large). Although visual analysis of child data for Triad 2 suggested more variable responding during caregiver-led sessions, overall effect sizes were similar across both student-led and caregiver-led sessions. In comparison, visual analysis and effect size measures indicated greater differences across contexts for Triad 1. Specifically, Remy’s mother Natalie (Triad 1) had the most limited increases in NDBI technique use (with mostly moderate effect sizes) and displayed high variability across sessions. In contrast, Remy’s student clinician data indicated large to very large effect sizes. The difference in adult behaviors corresponded to more limited effects on child outcomes for Triad 1 during caregiver-led sessions. Although Remy had very large effect sizes for total (prompted + unprompted) and unprompted spoken language responses for student clinician-led sessions, effect sizes were moderate for caregiver-led sessions. Given the scope of the project, future research should examine how the intervention affects children’s use of echolalia and whether adults respond differently to echolalia compared with other forms of child communication.

Compared with Triad 2, the more limited effects and overall variability for Triad 1 (Remy) may have been impacted by several factors. Throughout the study, Remy demonstrated fluctuations in emotion regulation (corresponding with behaviors such as yelling), which is common amongst autistic children (Conner et al., 2021). In view of this, variations in elicitation technique use may reflect clinically appropriate responsiveness rather than inconsistent implementation. For example, the clinical instructor provided Emma (the student clinician) with real-time coaching to support Remy’s engagement through techniques such as reducing verbal demands, simplifying verbal input, and incorporating sensory preferences. While aligned with NDBI principles such as following the child’s lead, matching the child’s energy (Frost et al., 2020) and making sure the child is in a happy, relaxed and engaged state (HRE; Gover et al., 2022), these adaptations may have reduced opportunities to create communication temptations. During periods of visible frustration or dysregulation, Emma appropriately prioritized maintaining the child’s assent to engage in play over his use of specific targeted responses. From a neurodiversity-affirming perspective, these adaptations may represent clinically meaningful responsiveness to child needs rather than a lack of intervention effectiveness. For Natalie (Remy’s mother), family cultural and linguistic practices may have also influenced intervention implementation. Throughout sessions, Natalie skillfully incorporated both French and English into interactions with her child. However, some of Natalie’s communication patterns, such as her observed use of directive language (e.g., “Can you give me…?” or “Can you help me…?”), may have reduced opportunities for child-led interaction and the creation of communication temptations. Natalie demonstrated strong strengths-based interaction patterns through frequent narration of Remy’s play and engagement with Remy’s interests; however, she frequently used nonspecific praise (e.g., ‘Remy est un très gentil garçon!’/Remy is a very good boy!) to reinforce Remy’s communication responses. This use of non-specific praise (rather than spoken language models that repeated, extended, expanded, or substituted Remy’s spoken responses) appeared to influence her response technique use, and subsequently Remy’s use of targeted communication responses. It should be noted that this mirrored a pattern of non-specific praise that has been previously documented within families from Hispanic backgrounds (McGuire et al., 2025).

### 4.1. Clinical Implications

Overall, the primary contribution of this study is not the cascading coaching model itself, which has been examined previously, but its application to autistic children who use echolalia and the explicit description of intervention adaptations designed to build upon children’s existing language repertoires. Existing intervention literature has largely focused on minimally speaking children or has not explicitly described how echolalic language can be incorporated into communication intervention planning. Accordingly, this study contributes preliminary evidence regarding the feasibility of extending NDBI coaching approaches to a population that remains underrepresented in intervention research.

Results support interpreting and building on echolalic children’s communicative attempts within meaningful contexts, as well as seeking to understand utterances and link them to the interaction (Ousley et al., 2024). This is notable given that prior research has emphasized reducing echolalia, which is often achievable but not consistently associated with improved communication quality (Blackburn et al., 2023; Neely et al., 2016). In contrast, these findings align with recent work showing that naturalistic, communication-focused approaches can improve engagement and functional language without requiring suppression of echolalia (Dinello & Gladfelter, 2025). Additionally, observed improvements in target child spoken communication may reflect growth in the complexity, specificity, and communicative utility of children’s spoken language rather than increases in verbal output alone (Lahey, 1988). Overall, the study adds to evidence that interventions should move beyond reduction goals and may instead position echolalia as a potential meaningful communicative resource, particularly within caregiver-implemented, naturalistic frameworks.

The multilevel outcomes observed across children, caregivers, and graduate student clinicians suggest that this approach has the potential to influence clinical service delivery, clinician training, and caregiver-mediated intervention. First, the findings provide preliminary evidence for the adaptation of NDBIs to align with the communication profiles and needs of speaking autistic children who use echolalia and their families. Adapted NDBIs provide a promising approach for embedding strengths-based, neurodiversity-affirming frameworks into intervention (Schuck et al., 2022; Wang et al., 2023). Importantly, when aligning NDBI with neurodiversity-affirming approaches, clinicians should also attend and respond to instances when autistic children do not demonstrate HRE (Gover et al., 2022). As NDBI technique use and child responsiveness are likely to vary more when a child’s assent-based learning state is prioritized, clinicians must use data collection to examine the aggregate effects of intervention over time (McGuire et al., 2025). Further individualized adaptations (e.g., embedding antecedent-based interventions into NDBI) for children and families should be considered when results remain stagnant, or children show consistent disengagement.

At a systems level, adult participant findings also highlight the importance of providing graduate student clinicians direct opportunities to practice supported caregiver coaching and NDBI techniques (Gevarter et al., 2025; McGuire et al., 2025; Nattress et al., in press). Additionally, findings provide preliminary evidence regarding the need for systems that allow for continued caregiver coaching for speaking autistic children who use echolalia. Although caregiver coaching is commonly embedded primarily in birth-three intervention programs (Friedman et al., 2012), as autistic children move beyond emerging communication and develop more advanced spoken language, caregiver support should not diminish. Rather, caregivers may benefit from learning new communication techniques that align with their child’s evolving communication profile and support ongoing language development. To achieve this, clinicians likely need to advocate for parent and family collaboration time.

In view of the varied outcomes of the multilingual and multicultural autistic children who use echolalia within this study, adaptations to NDBIs for echolalic language should be implemented in conjunction with other cultural and linguistic adaptations (Douglas et al., 2026). In the absence of research providing guidance on caregiving practices for families of autistic children from less-represented backgrounds (e.g., African immigrants to Western countries), clinicians should consider actively incorporating additional antecedent-based strategies that promote individualized, family-centered care. These may include, for example, motivational interviewing to obtain caregiver perspectives prior to intervention, brainstorming solutions to potential challenges to participation (e.g., transportation availability, child care, and language barriers), providing comprehensive information about intervention procedures and rationales prior to implementation, and actively soliciting caregiver perspectives and feedback throughout the intervention process (Benito-Gomez & Rojas, 2020; McGuire et al., 2025; Noh et al., 2026).

### 4.2. Limitations and Future Directions

While this pilot study provides important directions for adapting NDBI for echolalic autistic preschoolers, limitations related to measurement, sample size, intervention scope, and analytical depth highlight the need for continued research. Overall, addressing these areas will strengthen methodological rigor, enhance ecological validity, and support the development of more comprehensive and effective intervention models.

One limitation of the present study is the reliance on caregiver report and naturalistic observation to document echolalia use for child participant screening and inclusion. Although caregiver report is commonly used to capture caregiver experiences and perceptions in intervention research, reliance on caregiver report given the lack of standardized assessment tools for echolalia remains a broader methodological challenge in echolalia research. Although the present coding framework was developed for this study, its structure was informed by established language assessment traditions that examine language across form, content, and use (Lahey, 1988). Furthermore, Wilder and Redmond (2024) reported that Content/Form Analysis remains widely used among practicing speech-language pathologists in the United States. Nevertheless, the specific scoring system used in the present study has not been independently validated and should be considered preliminary. Future studies should explore application of existing language analysis procedures to echolalic speech, such as, but not limited to, weighted mean length of utterance (MLU), symbolic structure, utterance relevance, and more nuanced distinctions between prompted and unprompted responses (e.g., involving immediate or delayed echolalia).

Although multiple-probe designs are commonly used within single-case intervention research, the limited number of participant tiers reduces the strength of experimental control and should be considered when interpreting functional relations observed in the present study. With regard to design, although this study included multiple dependent variables across three participant groups and two triads, there were implementation factors that influenced design quality. First, although there were four total baseline phases and four total intervention phases across both adult and child outcomes, each design implementation only had two tiers. Given this structure and the presence of fewer than five baseline data points, the design would only meet What Works Clearinghouse (2022) standards for multiple probe designs with reservations. However, implementation-focused research suggests that two tiers may be sufficient (Lanovaz & Turgeon, 2020) and that the five-data-point requirement may limit feasibility in applied contexts (Harris et al., 2019). The caregiver training period was relatively brief (2 weeks), which may not have provided sufficient time to support skill acquisition, maintenance, or independent caregiver implementation. Additionally, maintenance data were not collected due to time constraints; future research should examine whether gains are maintained following intervention. Similarly, the study did not assess generalization to home or community settings. Because this study was conducted in a quiet clinical setting to support standardized implementation, future research should examine the approach in more naturalistic settings to assess its feasibility and effectiveness in everyday contexts. As a preliminary investigation, further research is needed to demonstrate reliable effects across participants, contexts, and time. Future work should extend the duration of training and incorporate a structured fading phase to support gradual caregiver independence, as well as incorporate generalization probes across settings (e.g., home environments).

Several methodological features strengthened the study’s internal validity. Although researchers did not ask participants to stop seeking other therapies outside of the intervention for ethical considerations, the multiple-probe across participants design provided experimental control through the staggered introduction of intervention following stable baseline performance and replication of effects across participants. While instrumentation also presented a potential threat, coding discrepancies were addressed through clarification of operational definitions and consensus among coders. Repeated measurement, procedural fidelity checks, and interobserver agreement further supported experimental control and measurement reliability.

Considerable variability was also observed across outcomes, including individual differences in adult NDBI technique use and child target spoken language communication responses. While this variability provides rich descriptive insight and helps contextualize findings, variability observed across measures (e.g., Remy’s varied outcomes) cannot be systematically examined within the current sample size. Additionally, although there was no pre-set criterion for mastery, child participation outcomes did not always meet the recommended rate of 1–2 communicative turns per minute (Coogle et al., 2015; Hamberger et al., 2022). It is important to note, however, that only targeted communicative response outcomes were measured, and the overall rate of children’s communicative turns (targeted and non-targeted) was likely higher.

Additionally, it should be noted that the iterative refinement of operational definitions and coding guidelines to achieve sufficient IOA for child-dependent variable measurement across participants and contexts revealed the methodological complexity of coding communicative behaviors in autistic children who use echolalia. With respect to child outcomes, although average IOA was generally high, the wider ranges for unprompted communication responses may suggest that these responses were more challenging to identify consistently across observers. At present, there is limited methodological guidance within the echolalia literature regarding the conceptualization, operationalization and coding of echolalic communicative acts within speech-language pathology and other disciplines (Stiegler, 2015). These challenges, as a result, may have contributed to initial coder discrepancies, highlighting the need for further development and standardization of observational measurement systems for echolalia-related communication outcomes.

The present study evaluated a multicomponent intervention package that included echolalia-responsive adaptations alongside clinician and caregiver coaching, performance feedback, intervention planning supports, and traditional NDBI practices. As a result, it is not possible to determine which components were responsible for observed changes in outcomes. Rather than examining the independent effects of echolalia-responsive adaptations, this study evaluated their integration within an established NDBI coaching framework. Thus, the present findings should be interpreted as evidence for the feasibility and potential promise of integrating echolalia-responsive practices within an NDBI coaching framework, rather than evidence of their independent effects. Future research using component analyses or comparative designs is needed to determine whether echolalia-responsive adaptations provide effects beyond established NDBI procedures.

Furthermore, interactional variables such as child engagement, regulation, and overall interaction quality that may influence adult and child outcomes were not explicitly addressed within the study. For instance, although the clinical instructor provided Remy’s student clinician with suggestions for re-engaging Remy or reducing intervention demands during moments where he appeared frustrated, adult participants were not explicitly taught how to assess whether a child was in an HRE state during sessions (Gover et al., 2022). Given prior applications of NDBIs to emotional regulation (Kushner et al., 2025) and evidence that autistic children often exhibit greater emotional intensity and reactivity than neurotypical peers (Day et al., 2023), research should expand NDBI adaptations to explicitly address sensory needs, engagement, and regulation. This includes integrating these elements into naturalistic instruction, co-planning with caregivers, and cascading coaching.

Expanding participant diversity is also critical, particularly to include more multilingual autistic children who use echolalia, as linguistic and cultural factors may meaningfully influence echolalic expression and function. While child and adult participants in this study reflected diverse racial, ethnic and linguistic backgrounds not often represented within ASD literature (Dinello & Gladfelter, 2025), additional considerations are necessary to ensure relevant cultural and linguistic adaptations (Douglas et al., 2026). Although researchers made some efforts to adapt the intervention to Remy’s family’s cultural and linguistic background (an African Canadian family speaking French, Yemba, English, and Dutch), including using French and Dutch/German in sessions, additional supports (e.g., assistance with transportation) and procedures (e.g., pre-intervention motivational interviewing) could have been considered. While several studies have examined cultural adaptations of NDBIs for Hispanic families (Douglas et al., 2026; McGuire et al., 2025), these approaches may not fully align with caregiving practices in other sociocultural contexts, including African families who have immigrated to Western countries. Although it is possible that some NDBI techniques used in this study were a cultural mismatch for Natalie (Remy’s mother), overall, a lack of research examining African immigrant families’ perspectives on autism (Munroe et al., 2016) limited the use of more proactive adaptations. Future research should expand cultural and linguistic adaptations of NDBIs to include underrepresented populations and examine how cultural and linguistic diversity intersects with echolalia. Additionally, qualitative research examining whether families from specific cultural backgrounds endorse elements of NDBI, considering the work of Benito-Gomez and Rojas (2020) and DuBay (2022), amongst others, is necessary.

Finally, although fidelity of coaching and adult participant use of specific NDBI techniques was measured in this study, these measures had some limitations. For instance, while student clinicians were instructed to provide caregivers with coaching consistent with the training they received, caregiver coaching was not directly observed or assessed in the present study. Within the coaching fidelity procedures, coaches were assessed based upon their use of feedback during and after play interactions (presence/absence). Thus, the quality of student clinician feedback provided to caregivers was not examined and represents an important area for future research. Additionally, although adult outcomes assessed a majority of techniques described in the NDBI Fidelity Rating Scale (NDBI-FI, Frost et al., 2020), adult participant use of other components (e.g., getting on the child’s level, using positive affect, following the child’s lead) was not explicitly measured. Furthermore, reflection, a common step within early intervention coaching models (Friedman et al., 2012), was not explicitly incorporated in coaching. In addition to teaching and assessing components identified in the NDBI-FI, further refinement of coaching fidelity should examine the quality and timing of in-session feedback, explore use of foundational NDBI techniques, and incorporate structured self-reflection tools for student clinicians and caregivers in order to strengthen effectiveness, rigor and replicability.

Overall, future investigations should extend this line of work through larger replications, direct measures of echolalia and communicative function, independent validation of communication outcome measures, and experimental evaluation of individual echolalia-responsive adaptations. Such work would help clarify both the mechanisms through which echolalia-informed practices operate and their potential contribution beyond existing NDBI procedures.

## 5. Conclusions

This pilot study provides preliminary evidence for NDBIs as a neurodiversity-affirming approach for autistic children who use echolalia. By recognizing the potential communicative value of echolalia and emphasizing responsive partner behavior, NDBIs offer a promising framework for supporting communication beyond early language goals. The findings suggest NDBIs can be adapted to promote advanced vocabulary, syntax, and communicative functions by building on children’s existing scripts, interests, and strengths. This approach aligns with perspectives that view echolalia as meaningful communication rather than a behavior to eliminate (Cohn et al., 2023a, 2023b; Dinello & Gladfelter, 2025). Future research using more rigorous experimental designs and larger, more diverse samples should examine the effectiveness, feasibility, and social validity of this approach across children and contexts to establish its efficacy. Nevertheless, the present study represents an important first step toward developing and evaluating strengths-based, echolalia-affirming intervention practices. Overall, these findings highlight the promise of approaches that support autistic communication styles while fostering language growth.

## Figures and Tables

**Table 1:** Child Participant Characteristics.

Child	Age	Race/ Ethnicity and Gender	Language Background	Diagnostic Status	VABS-III Age Equivalents	ADOS Echolalia-Related Items	
						A4. Immediate Echolalia	A5. Stereotyped/Idiosyncratic Use of Words or Phrases
Triad 1 Remy	4;11	African Canadian, Black, male	French, English, Yemba	Independent ASD diagnosis (3.5 y.o)	Receptive: 2;0 Expressive: 2;2 Interpersonal Relationships: 2;1	Echoing words and phrases regularly, but some spontaneous language, which can be stereotyped	Often uses stereotyped utterances or odd words or phrases, with some other language
Triad 2 Angel	4;6	Native American, Hispanic, male	English	Independent ASD diagnosis (3 y.o)	Receptive: 2;10 Expressive: 3;1 Interpersonal Relationships: 2;3	Occasional echoing	Use of words or phrases tends to be more repetitive than that of most individuals at the same level of expressive language, but not obviously odd

**Table 2:** Adult participant characteristics.

Adult	Age	Ethnicity and Gender	Language(s) Used	Highest Education Level	Job or Degree Program
Triad 1 Natalie (Remy’s caregiver)	39 y.o.	African Canadian, Black, Female	French, Dutch, English, Yemba	Master’s Degree	Full-time caregiver (Former Accountant)
Triad 1 Emma (Remy’s student clinician)	33 y.o.	White, female	English, German	BA; Master’s degree in progress	Second-year speech-language pathology graduate student
Triad 2 Naomi (Angel’s caregiver)	38 y.o.	Native American, Hispanic, female	English	Bachelor’s Degree	Registered Nurse
Triad 2 Liza (Angel’s student clinician)	33 y.o.	Asian Indian, White, Female	English	BA; Master’s degree in progress	First-year speech-language pathology graduate student

**Table 3:** Adult Targeted Temptations and Naturalistic Communication Contexts.

Adult Participant	Targeted Communication Temptations	Examples of Naturalistic Communication Contexts/Activities
Triad 1 Emma’s Targets (Remy’s Student Clinician)	Inadequate Portions (IP) Delayed Assistance (DA) Interrupting Routines (IR)	Balloon toy, MagnaTiles, Legos, pretend food, action figures, toy figurines, puppets, vehicles (e.g., cars, airplane, helicopter, etc.), trampoline, yoga ball, child-generated social routines (e.g., go to sleep)
Triad 1 Natalie’s Targets (Remy’s Mother)	Inadequate Portions (IP) Delayed Assistance (DA) Interrupting Routines (IR)	
Triad 2 Liza’s Targets (Angel’s Student Clinician)	Delayed Assistance (DA) Silly Situations (SS) Interrupting Routines (IR)	Balloon toy, light-up spinning toy, fine motor wind-up toys, dot paints, stickers, Play-Doh, bubbles, train track, car track, trampoline, owl number clock, Mr. Potato Head, Big Feelings Pineapple
Triad 2 Naomi’s targets (Angel’s Mother)	Inadequate Portions (IP) Delayed Assistance (DA) Silly Situations (SS)	

**Table 4:** Targeted Child Spoken Language Communication Responses.

Child Participant	Target Child Spoken Language Communication Responses (Targeted Across Temptations)			Example of Communication Temptation and Corresponding Child Language Targets
	Content/Vocabulary	Form/Syntax	Communicative Function/Verbal Operant	
Triad 1 Remy (Child)	Specific vocabulary, including early descriptors (i.e., color, size, quantity, texture, speed, physical state, etc.), verbs (i.e., crash, rock, run, bounce, kick, etc.), nouns (i.e., shapes, foods, etc.)	Verbally produce phrases (2–3 words) or simple sentences (4+) using simple syntax (i.e., Subject + Verb + Object)	Requesting assistance, rejecting assistance, repairing breakdowns, directing actions, negating, advocating for sensory needs	Temptation: SC holds a closed bag full of varied MagnaTiles (IP) ‘I want blue triangle.’ ‘I don’t want square.’
Triad 2 Angel (Child)	Specific vocabulary, including advanced descriptors (i.e., color, size, quantity, texture, speed, physical state, etc.), abstract verbs (i.e., know, remember, think, etc.), advanced nouns (i.e., idea, problem, etc.)	Verbally produce multi-word utterances (3+ words) using advanced syntax (i.e., conjunctions, prepositional phrases, noun phrases, verb phrases, etc.).	Expressing problems, expressing emotions, requesting assistance, rejecting assistance, repairing breakdowns, expressing wants/needs/ideas	Temptation: CG mislabels a body part on Mr. Potato Head (SS) ‘That’s not a nose, that’s teeth.’ ‘The eyes are missing’ ‘That’s not right’

Note: Student Clinician (SC), Caregiver (CG), Inadequate Portions (IP), Silly Situation (SS).

**Table 5:** Tau-U Effect Sizes for Adult Participant Implementation of NDBI Techniques.

	Adult Dependent Variables	Triad 1 Adult Participants				Triad 2 Adult Participants			
		Emma (SC)		Natalie (CG)		Liza (SC)		Naomi (CG)	
		Tau-U	*p*	Tau-U	*p*	Tau-U	*p*	Tau-U	*p*
NDBI Elicitation Techniques	Create Communication Temptation	1.0 (very large)	0.013 *	0.708 (large)	0.07	1.0 (very large)	0.013 *	0.958 (very large)	0.014 *
	Wait and Prompt	0.925 (very large)	0.021 *	0.6667 (large)	0.088	1.0 (very large)	0.013 *	1.0 (very large)	0.011 *
NDBI Response Techniques	Responsive Actions	0.963 (very large)	0.016 *	0.5833 (moderate)	0.136	1.0 (very large)	0.013 *	1.0 (very large)	0.012 *
	Responsive Language Models	0.778 (large)	0.052	0.5 (moderate)	0.201	1.0 (very large)	0.013 *	1.0 (very large)	0.012 *

Note. *p* values < 0.05 indicate statistical significance and are denoted by an asterisk (*).

**Table 6:** Tau-U Effect Sizes for Child Spoken Language Communication Responses Across Intervention Contexts.

Intervention Context	Targeted Child Communication Responses (Dependent Variables)	Remy (Triad 1)		Angel (Triad 2)	
		Tau-U	*p*	Tau-U	*p*
Student Clinician-Led Sessions	Total (Prompted + Unprompted) Spoken Language Responses	0.963 (very large)	0.0162 *	1.0 (very large)	0.0126 *
	Unprompted Spoken Language Responses	0.8889 (very large)	0.0265 *	0.7407 (large)	0.0645
Caregiver-Led Sessions	Total (Prompted + Unprompted) Spoken Language Responses	0.58 (moderate)	0.1356	1.0 (very large)	0.0105 *
	Unprompted Spoken Language Responses	0.50 (moderate)	0.2008	0.6667 (large)	0.0881

Note. *p* values < 0.05 indicate statistical significance and are denoted by an asterisk (*).

## Data Availability

Original data tables for the single case experimental design are available upon request to the corresponding author. Full interview transcriptions are unavailable due to privacy restrictions.

## Note

1 In view of the broad perspectives of the autistic community, authors consciously shift between person-first language (PFL; “person with autism”) and identity-first language (IFL; “autistic person”) to honor the variety of language perspectives and preferences (American Speech-Language-Hearing Association, n.d.).

## Supplementary Materials

The following supporting information can be downloaded at: https://www.mdpi.com/article/10.3390/bs16091553/s1, Figure S1: Instructional Visual Aid; Figure S2: Example of Blank Activity Planner; Figure S3: Coaching Checklist for Sessions 1–3 and Sessions 4–6; Supplemental Material S4: Refined Caregiver Echolalia Questionnaire.
