# Supplementary material for: Adapting NDBIs for Autistic Children Who Use Echolalia: A Cascading Coaching Approach to Supporting Communication and Language Development
Source: Behav Sci (Basel). 2026 Sep 2;16(9):1553. doi: 10.3390/bs16091553 (PMC13603362; doi:10.3390/bs16091553)
Supplement: Supplementary file 1 [file behavsci-16-01553-s001.zip › Figure S1. Instructional Visual Aid.pdf]

Creating Communication

CREATE opportunities for the child to respond

WAIT for the child to respond

PROMPT a desired response

REINFORCE the child's communication

AND **LABEL** what they request, refuse or comment about

Let the child **ENGAGE** with materials

AND **LABEL** what they request, refuse or comment about
