# Supplementary material for: Adapting NDBIs for Autistic Children Who Use Echolalia: A Cascading Coaching Approach to Supporting Communication and Language Development
Source: Behav Sci (Basel). 2026 Sep 2;16(9):1553. doi: 10.3390/bs16091553 (PMC13603362; doi:10.3390/bs16091553)
Supplement: Supplementary file 1 [file behavsci-16-01553-s001.zip › Figure S2. Example of Blank Activity Planner.pdf]

Child's Initials: ____________________ Graduate Clinician: ______________________________ Date: ____________________

Activity Planner

Project SCENES

Target Temptation	Activities	Types of Responses	Wait Time (at least 3 seconds)	Prompt	Respond & Label	Let the child engage with materials & Label
					Reinforce<br><br>Label - Use a contextually relevant word/phrase such as:	Engage with Materials<br><br>Label - Use a contextually relevant word/phrase such as:
					Reinforce<br><br>Label - Use a contextually relevant word/phrase such as:	Engage with Materials<br><br>Label - Use a contextually relevant word/phrase such as:
					Reinforce<br><br>Label - Use a contextually relevant word or phrase such as:	Engage with Materials<br><br>Label - Use a contextually relevant word/phrase such as:
