# Supplementary material for: Adapting NDBIs for Autistic Children Who Use Echolalia: A Cascading Coaching Approach to Supporting Communication and Language Development
Source: Behav Sci (Basel). 2026 Sep 2;16(9):1553. doi: 10.3390/bs16091553 (PMC13603362; doi:10.3390/bs16091553)
Supplement: Supplementary file 1 [file behavsci-16-01553-s001.zip › Figure S3. Coaching Checklist for Sessions 1–6 .pdf]

Coaching Checklist 1-3

Project SCENES Summer Autism Clinic

Before Play Observation:

	Discuss	Model	Add to planner together
Communication Temptation			
Routines			
Responses			
Wait Time			
Prompting			
Reinforce/label			
Engage/label			
**Ask for Questions**			
Coach makes available: Activity Planner, Visual Aid & Timer			

During Play Observation:

**15-minute play session**	Preferred items available
Coach provides **verbal cues & assistance as needed** during playtime	
**Timer and visual aid available**	

After Play Observation:

	Provide feedback on **at least 3 strategies**
Create	
Wait	
Prompt	
Reinforce/label	
Engage/label	

Coaching Checklist 4-6

Project SCENES Summer Autism Clinic

Before Play Observation:

Coach makes available	**Activity planner**
	**Visual aid**
	**Timer**
**Ask for Questions**	
Model strategies if needed	

During Play Observation:

**15-minute play session**	Preferred items available
Coach provides **verbal cues & assistance as needed** during playtime	

After Play Observation:

	Provide feedback on **at least 3 strategies**
Create	
Wait	
Prompt	
Reinforce/label	
Engage/label	
