# Supplementary material for: Adapting NDBIs for Autistic Children Who Use Echolalia: A Cascading Coaching Approach to Supporting Communication and Language Development
Source: Behav Sci (Basel). 2026 Sep 2;16(9):1553. doi: 10.3390/bs16091553 (PMC13603362; doi:10.3390/bs16091553)
Supplement: Supplementary file 1 [file behavsci-16-01553-s001.zip › Supplemental Material S4.pdf]

Supplemental Material 4

Refined Caregiver Echolalia Questionnaire

Student Clinician/Caregiver Echolalia Information

1. What is your level of echolalia knowledge? *Circle one or more.*
    a. I feel very knowledgeable about echolalia.
    b. I feel somewhat knowledgeable about echolalia.
    c. I feel slightly knowledgeable about echolalia.
    d. I do not feel knowledgeable about echolalia.
    e. I have not had the opportunity to learn about echolalia.
2. What resources have you used to learn about echolalia?[1,2]
    a. Professional training (online courses, training from private SLP, information session from school, information session from disability organization)
    b. Self-taught knowledge (YouTube videos, podcasts, books, online search, friendship discussion groups, online communities)
    c. Knowledge through experience of person with echolalia
    d. No current knowledge due to ______________________

Child Echolalia History

1. Have you as a parent heard the term "echolalia" before? Where? When?

    ____

2. Is your child able to talk using single words?
    a. Never
    b. Sometimes
    c. Often
    d. Always
3. Is your child able to talk using short sentences?[3]

a. Never
b. Sometimes
c. Often
d. Always

4. Can you have a conversation/dialogue with your child by taking turns? In other words, you talk, child talks, you ask, child answers, child asks, you answer.[3]
    a. Never
    b. Sometimes
    c. Often
    d. Always
5. Does the child imitate phrases spoken by others?[3]
    a. Never
    b. Sometimes
    c. Often
    d. Always
6. How long after their production? For example, they heard today and repeated tomorrow, in a week, in a month, several months later?[3]
    a. Immediate
    b. Next day
    c. Next week
    d. Next month
7. Does your child say the same thing several times and in the same way?[3]
    a. Never
    b. Sometimes
    c. Often
    d. Always

8. Does the child say phrases they heard someone else say exactly the same way it was spoken?[3]

a. Never
b. Sometimes
c. Often
d. Always

9. Does the repetition of these phrases by the child occur for no apparent reason?[3]

a. Never
b. Sometimes
c. Often
d. Always

10. Does the repetition of these phrases by the child appear to serve a purpose?

a. Never
b. Sometimes
c. Often
d. Always

11. Why do you think your child repeats these phrases?

____

12. Is the repetition of phrases usually about a specific subject?[3]

a. Never
b. Sometimes
c. Often
d. Always

13. Does repetition of phrases occur in specific moments? For example, when the child is angry, isolated, afraid, etc.[3]

a. Never
b. Sometimes

c. Often

d. Always

14. How often is this repetition (echolalia) happening? For example, several times an hour, day, week, month, etc.[3]

____

15. When did the repetition of speech of another first occur? What was the child's age?

____

16. Did you observe this repetition of speech of another changing over time? How?[3]

a. Did you observe a **decrease** in the frequency of repetitions over time?[3]

i. Yes

ii. No

iii. Sometimes

b. Did you observe an **increase** in the frequency of repetitions over time?[3]

i. Yes

ii. No

iii. Sometimes

c. Did you observe a change in the **types** of repetitions over time?[3]

i. Yes

ii. No

iii. Sometimes

d. Did you observe a change in the **structure** of repetitions over time?[3]

i. Yes

ii. No

iii. Sometimes

e. Did you observe a change in the **use** of repetitions over time?[3]

i. Yes

ii. No

iii. Sometimes

Adapted From:

1. Cohn, E. G., McVilly, K. R., & Harrison, M. J. (2023a). Echolalia as defined by parent communication partners. Autism & Developmental Language Impairments, 8, 23969415231151846. https://doi.org/10.1177/23969415231151846
2. Cohn, E. G., McVilly, K. R., & Harrison, M. J. (2023b). Mapping the parent experience of echolalia in autism spectrum disorder onto a conceptual taxonomy. *International Journal of Speech-Language Pathology, 26*(6), 793–808. https://doi.org/10.1080/17549507.2023.2244201
3. Mergl, M., & Azoni, C. A. S. (2015). Echolalia's types in children with autism spectrum disorder. *Revista CEFAC, 17*(6), 2072–2080. https://doi.org/10.1590/1982-021620151763015
